# Roniciclib down-regulates stemness and inhibits cell growth by inducing nucleolar stress in neuroblastoma

**DOI:** 10.1038/s41598-020-69499-6

**Published:** 2020-07-31

**Authors:** Marzia Ognibene, Annalisa Pezzolo

**Affiliations:** 1 Laboratorio Cellule Staminali Post Natali e Terapie Cellulari, IRCCS Istituto Gaslini, 16147 Genova, Italy; 2 Present Address: Unità di Genetica Medica, IRCCS Istituto Gaslini, 16147 Genova, Italy

**Keywords:** Cancer, Cell biology, Drug discovery, Molecular biology, Stem cells, Oncology

## Abstract

Neuroblastoma, an embryonic tumor arising from neuronal crest progenitor cells, has been shown to contain a population of undifferentiated stem cells responsible for the malignant state and the unfavorable prognosis. Although many previous studies have analyzed neuroblastoma stem cells and their therapeutic targeting, this topic appears still open to novel investigations. Here we found that neurospheres derived from neuroblastoma stem-like cells showed a homogeneous staining for several key nucleolar proteins, such as Nucleolin, Nucleophosmin-1, Glypican-2 and PES-1. We investigated the effects of Roniciclib (BAY 1000394), an anticancer stem cells agent, on neurospheres and on an orthotopic neuroblastoma mouse model, discovering an impressive inhibition of tumor growth and indicating good chances for the use of Roniciclib in vivo. We demonstrated that Roniciclib is not only a Wnt/β-catenin signaling inhibitor, but also a nucleolar stress inducer, revealing a possible novel mechanism underlying Roniciclib-mediated repression of cell proliferation. Furthermore, we found that high expression of Nucleophosmin-1 correlates with patients’ short survival. The co-expression of several stem cell surface antigens such as CD44v6 and CD114, together with the nucleolar markers here described, extends new possibilities to isolate undifferentiated subpopulations from neuroblastoma and identify new targets for the treatment of this childhood malignancy.

## Introduction

Neuroblastoma (NB) is an embryonic pediatric tumor that originates from neural crest progenitors cells^1,2^. NB is the most common tumor in young children, about the 90% of cases happen in children less than 5 years old^3^. NB is characterized by clinical, biologic and genomic remarkable heterogeneity that affected the survival of NB patients despite intensive therapy^4,5^.

Cancer stem cells (CSCs) are suggested to be responsible for drug resistance and relapse due to their ability to self-renew, and to differentiate into heterogeneous lineages of cancer cells^6^. Heterogeneity within a given cancer arises from diverse cell types recruited to the tumor, and from genetic or epigenetic differences among the cancer cells themselves^7,8^. CSCs are the exclusive sources of all tumor cells, survive and persist after cancer therapy and are responsible for tumor relapse and metastasis^9^. The concept of CSCs has immediate therapeutic consequences: if cancer growth is sustained by CSCs, then curative therapy will require targeting of this specific sub-population^10^. The clinical presentation and the treatment response of advanced NB, which results in relapse and a refractory state after a good response to the initial chemotherapy, suggests the possibility that CSCs exist in NB tumors^11^. Moreover, the heterogeneity of NB tumor histology and biology may suggest the existence of self-renewing multipotent CSCs^12^. NB tumors with unfavorable prognosis have been shown to contain a population of undifferentiated stem cells responsible for their malignant state^13^. Putative CSCs identification in primary tumor sphere derived from NB patients, and from cell lines was reported previously^14–21^. In this respect, our group showed that the Frizzled receptor 6 (Fzd-6) is a new surface marker of aggressive NB cells with stem cell-like features^22^. Fzd-6^+^ NB cells formed neurospheres with high efficiency, resistant to doxorubicin killing, and expressing high levels of mesenchymal markers such as Twist-1 and Notch-1^22^. These data suggested that targeting CSCs signatures might affect chemo-resistant NB cells. Shohet’s group defined an highly tumorigenic NB cell subpopulation, isolated from primary tumors and from NB cell lines, with stem cell characteristics that expressed the receptor for granulocyte colony stimulating factor CD114^23^. Recently, it has been described that nucleolin (NCL) is likely to be a salinomycin-binding target, and a crucial regulator involved in NB CSCs activity^24^. NCL is a nucleocytoplasmic protein, abundantly expressed in the nucleolus, involved in various cellular processes including ribosome biogenesis and chromatin structure^25^. Because of its selective expression on the surface of different cancer cells, but not on their normal counterparts, NCL represents an appealing target for antitumor treatments^26,27^. Moreover, NCL is one of the nucleolar stress-related proteins that translocate from the nucleolus to the nucleoplasm and/or cytoplasm during nucleolar stress^28^. Recently, novel functions, independent from ribosome biogenesis, have been attributed to nucleolus as a regulator of cell cycle progression^29^, genome stability^30^, telomere maintenance^30^, nuclear architecture^31^, senescence^32^, apoptosis^33^, and stress responses^34^. Nucleolar stress leads to down-regulation of the nucleolar functions and to some biological cell behavior changes, including disorders of protein translation, cell cycle arrest, and cell death^35^. Furthermore, alterations in the nucleolar functions have been associated with multiple forms of cancer^36–38^.

Roniciclib (BAY 1000394), a cyclin dependent kinase inhibitor, has been recently identified as a potent therapeutic benefit for high-risk NB through the targeting of the CSCs^39^. Here we tried to test its effects on stemness markers expression, nucleolar stress, neurospheres formation ability and tumor growth in a preclinical mouse model of NB. Although the above studies have paved the way to the identification of NB CSCs and their therapeutic elimination, additional work has demonstrated that this topic is still open to novel investigations and discoveries. Moreover, the action mechanisms of Roniciclib are not yet completely understood.

This study proposes the involvement of several key nucleolar proteins, such as NCL^25–28^, Nucleophosmin-1 (NPM1)^40^, Glypican-2 (GPC2)^41^, and Pescadillo Ribosomal Biogenesis Factor-1 (PES1)^42^ in the nucleolar stress induced by Roniciclib in both stem and non-stem NB cells. All of them may help the development of new therapeutic approaches, strategically inducing nucleolar stress in NB cells as a potential anti-tumor therapy.

## Results

### Morphological and molecular characterization of neuroblastoma tumor spheres

**Figure 1:**
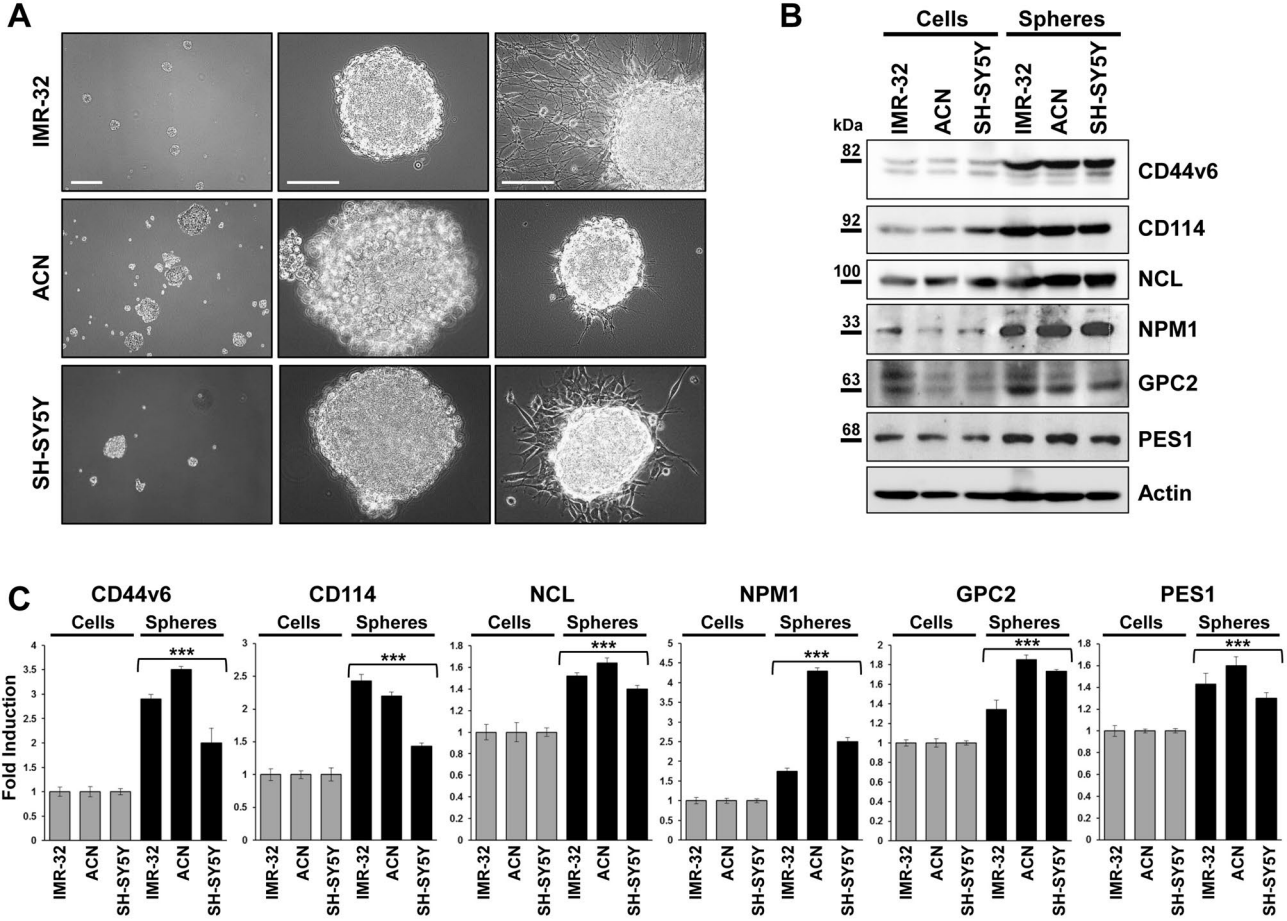
Neuroblastoma tumor spheres show high levels of cancer stem cell surface markers and of key nucleolar proteins. (**A**) Neurospheres derived from IMR-32, ACN and SH-SY5Y neuroblastoma cell lines cultured for three days in serum-free medium and in non-adherent conditions. Spheres show differences in number and dimensions (left photos), cells aggregation (middle photos) and adhesion ability (right photos). (Scale bars: 200 µm on the left and 100 µm in the middle and on the right). (**B**) Protein lysates from IMR-32, ACN and SH-SY5Y cell lines and from neurospheres derived by each cell line were collected and subjected to Western blot analysis with anti-CD44v6, anti-CD114, anti-Nucleolin (NCL), anti-Nucleophosmin-1 (NPM1), anti-Glypican-2 (GPC2) and anti-Pescadillo Ribosomal Biogenesis Factor-1 (PES1) antibodies. Cropped blots are shown here, and black lines indicate where one part of the blot ends and another begins. Supplementary Figure S5 shows the entire blots images. (**C**) Neurospheres protein levels were quantified by densitometry, normalized to those of each cell line (fold induction = 1) and to the content of the loading control protein (Actin), then visualized by histograms. Data are representative of three independent experiments ± SD (*** *p* < 0.001).

**Figure 2:**
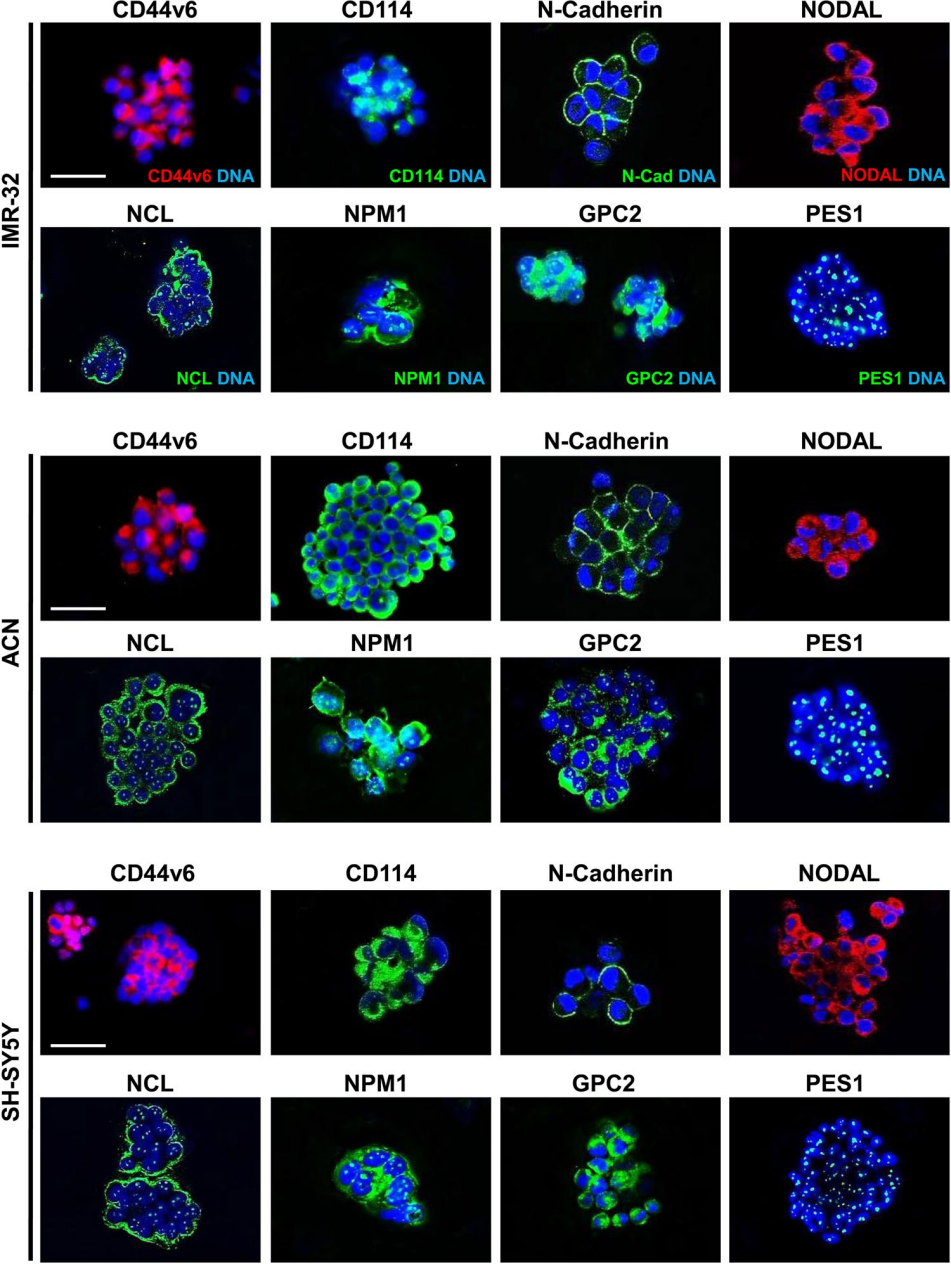
Cell localization of cancer stem cell surface markers and of nucleolar proteins in neuroblastoma tumor spheres. Immunofluorescence analysis of neurospheres derived by IMR-32, ACN and SH-SY5Y cell lines collected after three days of culturing in serum-free medium, using anti-CD44v6 (red), anti-CD114 (green), anti-N-Cadherin (green), anti-Nodal (red), anti-NCL (green), anti-NPM1 (green), anti-GPC2 (green) and anti-PES1 (green) antibodies. Cells were counterstained with DAPI to visualize nuclei (blue). (Scale bars: 200 µm).

We produced serial tumor spheres using three NB cell lines to enrich in undifferentiated stem-like cells and assess their self-renewal potential and other stemness features. We selected IMR-32, ACN and SH-SY5Y cell lines due to their different genomic characteristics: IMR-32 display *MYCN* (v-myc myelocytomatosis viral related oncogene, neuroblastoma derived) amplification, whereas ACN and SH-SY5Y do not, and SH-SY5Y exhibit strong c-MYC protein expression. IMR-32, ACN and SH-SY5Y cells efficiently produced serial neurospheres under serum free medium conditions. Furthermore, primary neurospheres enzymatically digested after 5–7 days of culture and re-plated as single-cell suspension, generated a second and third passage of spheres (self-renew), a feature that has been associated to CSCs^10^. We observed morphological variability among the neurospheres formed by the three different NB cell lines. Neurospheres formed by IMR-32 cells are numerous, stable, with regular shape but small in size (80–100 µm in diameter, on average) and, if transferred on adherent supports, they rapidly take strict contact to the plate by protrusion of long and branched neurite-like extensions. Neurospheres formed by ACN cells are numerous, large in size (200–250 µm in diameter, on average) with not perfectly spherical shape, constituted by mildly aggregated cells, and weakly adherents. Finally, neurospheres formed by SH-SY5Y are few, medium in size (100–150 µm in diameter, on average), with regular shape, very stable and able to strongly adhere to the substratum (Fig. 1A). We examined the presence and amounts of specific protein markers of stemness on neurosphere derived from NB cell lines by Western blot (Fig. 1B, C) and immunofluorescence analysis (Fig. 2; see the immunofluorescence analysis of the adherent parental cell lines in Fig. 6, further into the text). All tumor spheres expressed the variant 6 isoform of adhesive receptor CD44 (CD44v6), a cell surface protein expressed in CSCs of several cancer types but not in somatic cells^43–46^. CD44 exists as a large family of isoforms, produced by the alternative splicing of up to 20 exons, and CD44v6, in particular, is required for CSCs migration and generation of metastatic tumors^44^. Tumor spheres co-expressed other NB stem cells marker proteins such as CD114^23,24^ and NCL^25^ and the nucleolar antigens NPM1^40^ and PES1^42^, as well as GPC2, that is an oncoprotein strongly candidate to be an immunotherapeutic target in NB^41^. While studying embryonic antigens in neurospheres, we discovered that they expressed the stem cell marker N-Cadherin and the embryonic morphogen Nodal (Fig. 2). Interestingly, Nodal is a member of the transforming growth factor beta super-family, and it is a critical factor involved in normal embryonic development including maintenance of pluripontency in human embryonic stem cells^47^.

### Association of *NPM1* gene expression with neuroblastoma patients’ outcome

We evaluated the association of the expression of the analyzed stemness markers with stage 4 NB patients’ outcome. Using the neuroblastoma Kokac^48^ public patients’ data-set from the R2 Genomics Analysis and Visualization Platform (http: r2.amc.nl), we obtained online microarray analysis results of the RNA sequencing dataset (n = 649 tumors) with available survival endpoints. The most appropriate cut-off for the survival analyses was established at the expression value where the separation of survival curves reached the maximum (log-rank test). We found that high expression of *NPM1* gene, in particular (probe-set A_24_P188941, chosen for the highest average signals for analysis), was significantly associated with worse event-free and overall survival of stage 4 NB patients, consistently across the dataset (Supplementary Fig. S1).

### CD44v6^+^ cells identified in primary NB samples are enriched by chemotherapy

We have performed experiments aimed at testing the expression of CD44v6 in primary NB. NB tissue sections samples from 13 patients at stage 4 of disease were tested for CD44v6 expression by immunofluorescence (Supplementary Fig. S2A, panel 1). We consistently detected a rare population of cells expressing the antigen CD44v6 (range 0.5 ± 0.1%-1.9 ± 0.1%) in every primary human biopsy specimen obtained before chemotherapy (Supplementary Table S1). Curiously, the human NB cell lines IMR-32, ACN and SH-SY5Y showed a high amount of CD44v6^+^ cells with a percentage of about 40% on average. Since we hypothesize that CD44v6^+^ cells may represent a chemo-resistant subpopulation of NB, as it is for CSCs populations in other tumors^43–46^, we therefore analyzed CD44v6 expression from the same primary human NB samples with high risk disease resected after rapid COJEC chemotherapy treatment^49^. These patients carrying chemotherapy resistant tumors had very poor prognosis^50^. The percentage of CD44v6^+^ cells, that ranged from 0.5 ± 0.1% to 1.9 ± 0.1% at diagnosis, increased around 5- to tenfold in NB samples obtained from patients who had received chemotherapy (range 5 ± 3% to 19% ± 2%; *p* < 0.001) (Supplementary Table S1 and Supplementary Fig. S2A, panel 2). These findings supported the working hypothesis. Furthermore, metastatic bone marrow aspirate from a multi-relapsed patient with stage 4 NB was found to contain 8% of CD44v6^+^ cells (Supplementary Fig. S2A, panel 3). These observations suggest that CD44v6 antigen may be candidate for the development of CSCs-targeting therapies for refractory NB patients.

### Identification of CD44v6^+^ tumor cells in an orthotopic mouse model of human neuroblastoma

In subsequent experiments, IMR-32 cells were injected in the adrenal gland capsule of five nude mice to generate orthotopic tumors recapitulating the natural history of primary human NB. The choice of the IMR-32 cell model was based on previous studies from our group^51^. In analogy to what we observed with primary NB, orthotopic tumors were found to contain a little population of CD44v6^+^/CD114^+^ cells predominantly in perivascular spaces, where CSCs usually reside^52^ (Supplementary Fig. S2B). When we wondered if the expression of *CD44v6* and *CD114* genes can be associated with stage 4 NB patients’ outcome, consulting public data-sets from the R2 Genomics Analysis and Visualization Platform we found that *CD114* gene resulted not sufficiently significant, while *CD44v6* gene does not exist in none database. This is possibly because these two stemness markers seem to be little expressed at the onset, but they increase especially after chemotherapy^23^.

### Roniciclib induces growth arrest, cell differentiation, impairment of neurospheres formation ability, and down-modulates stemness-related markers in NB cells

**Figure 3:**
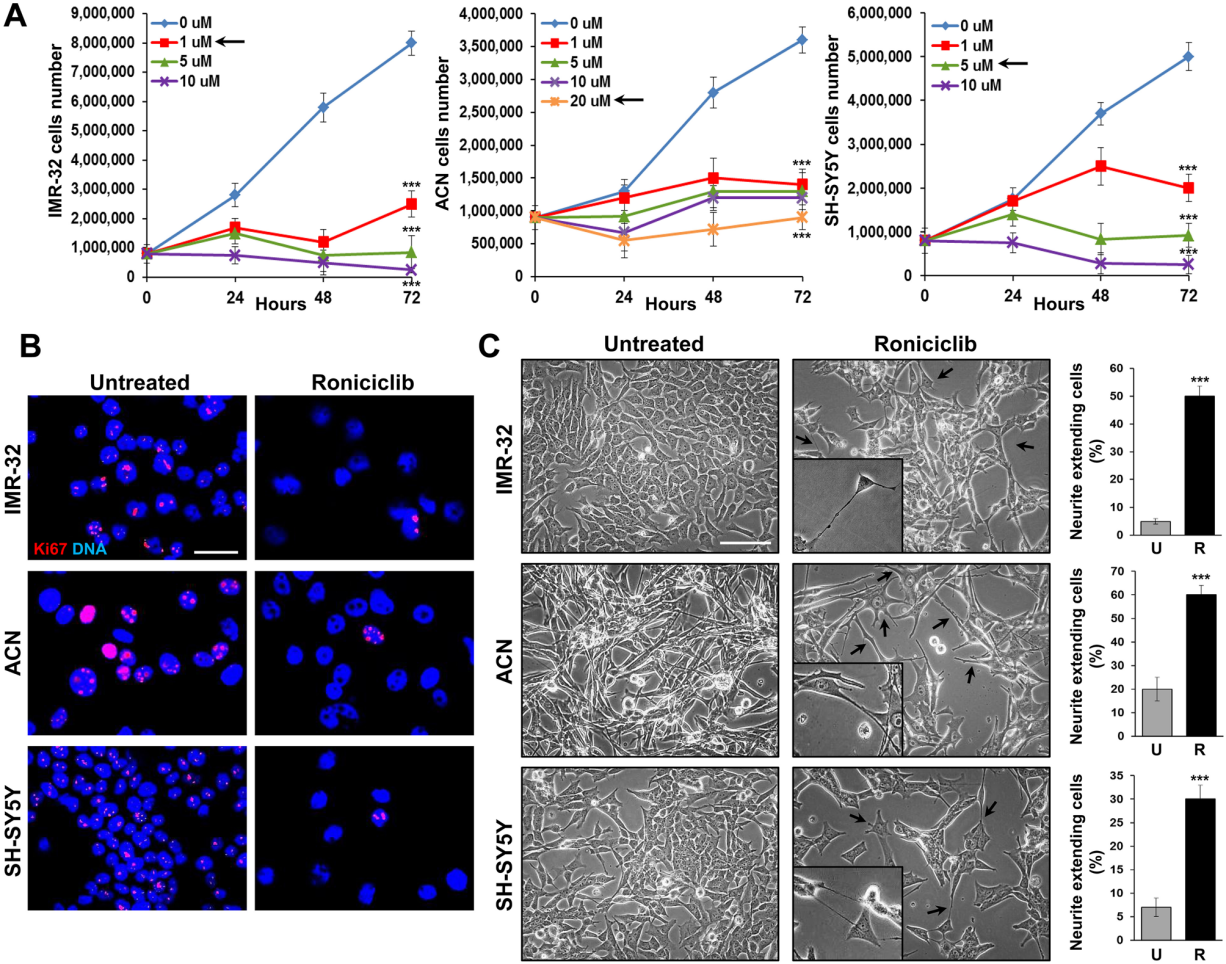
Roniciclib induces growth arrest and cell differentiation in neuroblastoma cells. (**A**) IMR-32, ACN and SH-SY5Y neuroblastoma cell lines were cultured in presence of various concentrations of Roniciclib for 24, 48 and 72 h. At each harvest point, cells were trypsinized and counted in Trypan blue. Untreated cells (Roniciclib 0 µM) were cultured with 0.1% DMSO. Arrows indicate the Roniciclib concentrations chosen for each cell line after 72 h, which gave significant effects without toxicity. Data are representative of three independent experiments ± SD (*** *p* < 0.001). (**B**) Proliferation rate evaluated by immunofluorescence analysis of IMR-32, ACN and SH-SY5Y neuroblastoma cell lines untreated (Roniciclib 0 µM) or treated with Roniciclib 1 µM, 20 µM and 5 µM respectively for 72 h, using the anti-Ki-67 (red) antibody. Cells were counterstained with DAPI to visualize nuclei (blue). (Scale bar: 20 µm). (**C**) Morphological characteristics of IMR-32, ACN and SH-SY5Y cell lines untreated (Roniciclib 0 µM) or treated with Roniciclib 1 µM, 20 µM and 5 µM respectively for 72 h. Roniciclib treated cells displayed a reduced growth rate, increase in cell size, elongated shape and emission of neurite-like extensions (arrows) (Scale bar: 40 µm). In the insets are visualized enlargements of particular examples of neurite-like protrusions produced by each cell line. Histograms represent the percentages of cells extending neurite-like protrusions among untreated (U) or Roniciclib treated (R) cells after 72 h. Data are representative of three independent experiments ± SD (*** *p* < 0.001).

**Figure 4:**
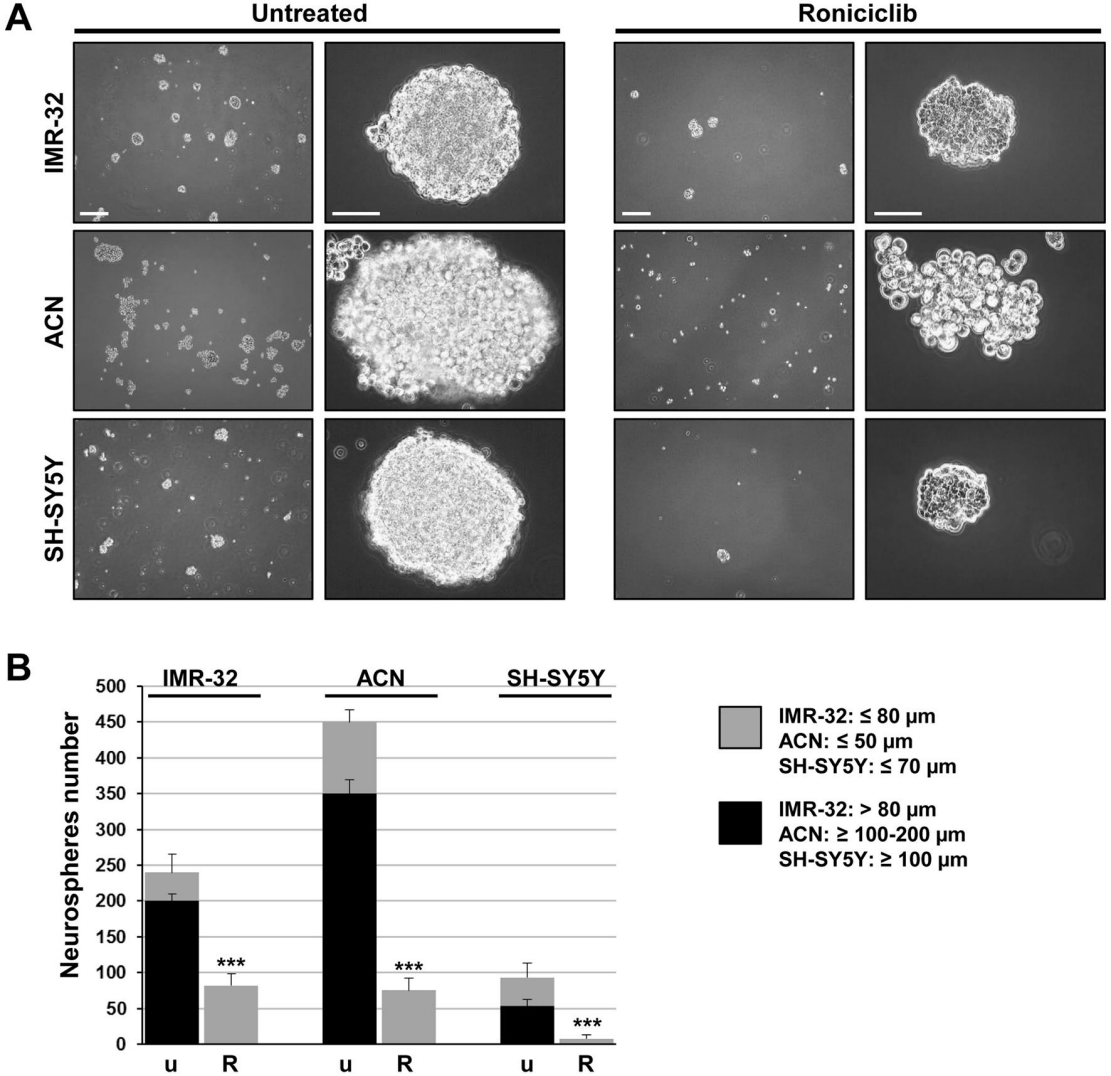
Roniciclib impairs neuroblastoma neurospheres formation ability. (**A**) Microscopic analysis of neurospheres produced by IMR-32, ACN and SH-SY5Y cells untreated (Roniciclib 0 µM) or treated with Roniciclib 1 µM, 20 µM and 5 µM respectively, for 72 h, and cultured for three days in serum-free medium maintaining the same Roniciclib concentrations. (Scale bars: 200 µm on the left of both panels; 100 µm on the right of the untreated spheres panel and 40 µm on the right of the Roniciclib treated spheres panel). (**B**) Average diameter of neurospheres produced by untreated IMR-32, ACN and SH-SY5Y cells (u) and by Roniciclib treated cells (R). Data are representative of three independent experiments ± SD (*** *p* < 0.001).

We analyzed the effects of Roniciclib on IMR-32, ACN, and SH-SY5Y cell lines. Roniciclib (BAY 1000394) is a cytotoxic agent directed against cell cycle and cyclin-dependent kinases^53^ that inhibits NB stem cells growth *in vitro*^39^. Neuroblastoma cell lines were cultured in presence of various concentrations of Roniciclib for 24, 48 and 72 h, while control cells were cultured in equal percentage of DMSO (0.1%). At each harvest point, cells were trypsinized and counted in Trypan blue to test their viability. We chose for each cell line the Roniciclib concentration that gave significant growth effects without toxicity after 72 h: 1 µM for IMR-32, which showed a higher sensitivity, 20 µM for ACN and 5 µM for SH-SY5Y (Fig. 3A). We observed an evident cell growth arrest induced by Roniciclib, assessed by Ki-67 staining, which identifies proliferating cells: as expected, treated cells had significantly fewer positive nuclei than the untreated ones (Fig. 3B). Roniciclib caused morphological changes in all cell lines, inducing increase in cell size and elongated or even star-like shapes (see ACN cells), with emission of neurite-like protrusions too, as sign of higher cell differentiation^54^. The percentage of neurite-like extensions showed by Roniciclib-treated cells was higher than that showed by untreated cells (Fig. 3C). After Roniciclib treatment, cells were able to produce only little, dark and weakly aggregated neurospheres, that rapidly unraveled, especially if handled, and we detected a pronounced impairment in neurospheres formation ability, consistently observed over the entire course of the experiments (Fig. 4A). After three days in serum-free medium, we counted the formed spheres and we found that Roniciclib reduced the number of neurospheres with stable renewal ability, achieving 68% less than control for IMR-32, 84% less than control for ACN, and 90% less than control for SH-SY5Y, on average. Smaller spheres were mostly detectable after Roniciclib treatment, rather than among the ones produced by untreated cells (Fig. 4B).

**Figure 5:**
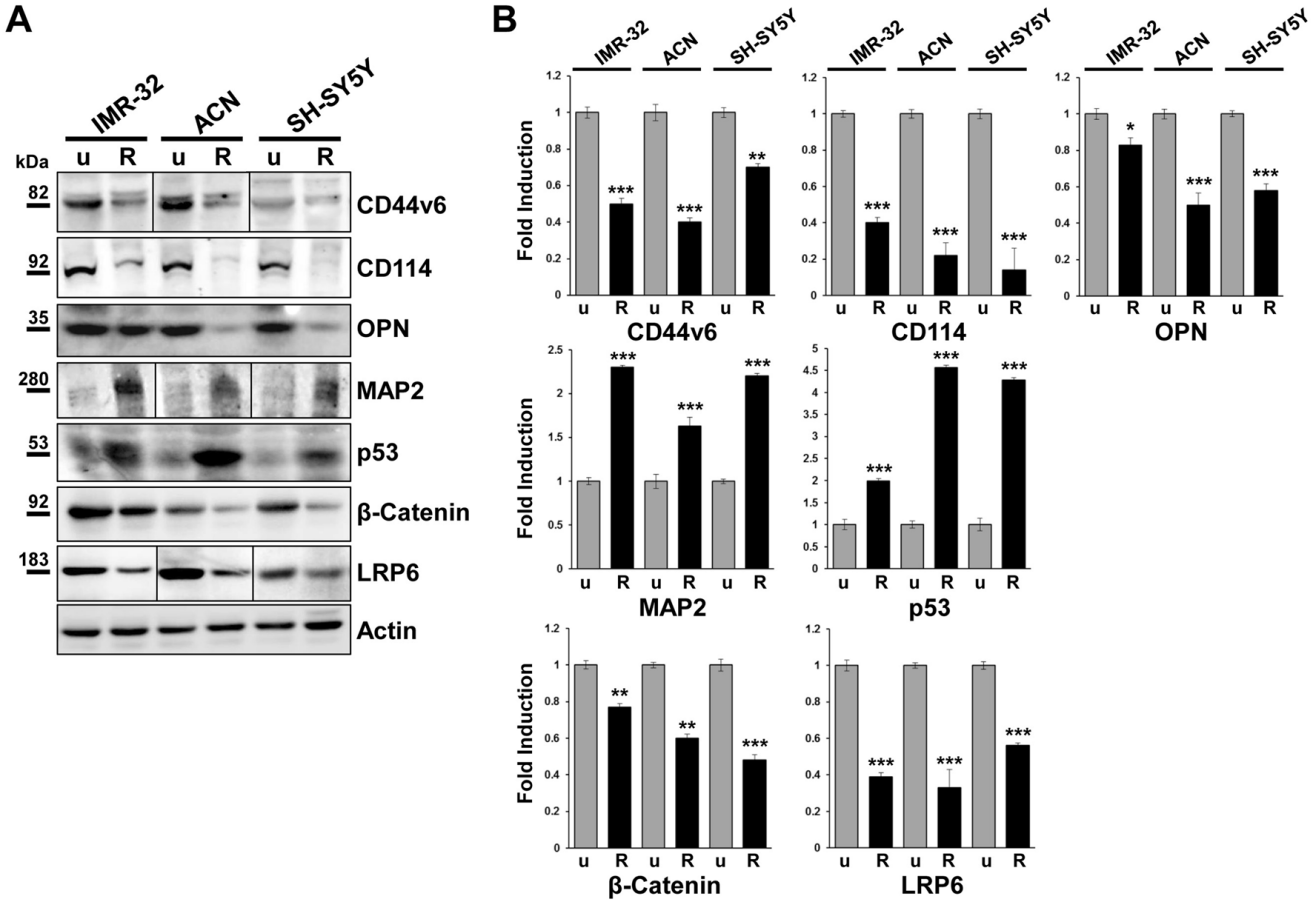
Roniciclib strongly inhibits cancer stem cells markers expression while enhances p53 onco-suppressor level in neuroblastoma cells. (**A**) Protein lysates from IMR-32, ACN and SH-SY5Y cell lines untreated (u = Roniciclib 0 µM) or treated with Roniciclib (R) 1 µM, 20 µM and 5 µM respectively, for 72 h were collected and subjected to Western blot analysis with anti-CD44v6, anti-CD114, anti-Osteopontin (OPN), anti-Microtubule-associated protein-2 (MAP2), anti-p53, anti-β-catenin and anti-Low density lipoprotein related protein (LRP6) antibodies. Cropped blots are shown here, and black lines indicate where one part of the blot ends and another begins. Supplementary Figure S6, panels 1–2, shows the entire blots images. (**B**) Protein levels of the Roniciclib treated cells were quantified by densitometry, normalized to those of the untreated cells (fold induction = 1) and to the content of the loading control protein (Actin), then visualized by histograms. Data are representative of three independent experiments ± SD (* *p* < 0.05; ** *p* < 0.01; *** *p* < 0.001).

**Figure 6:**
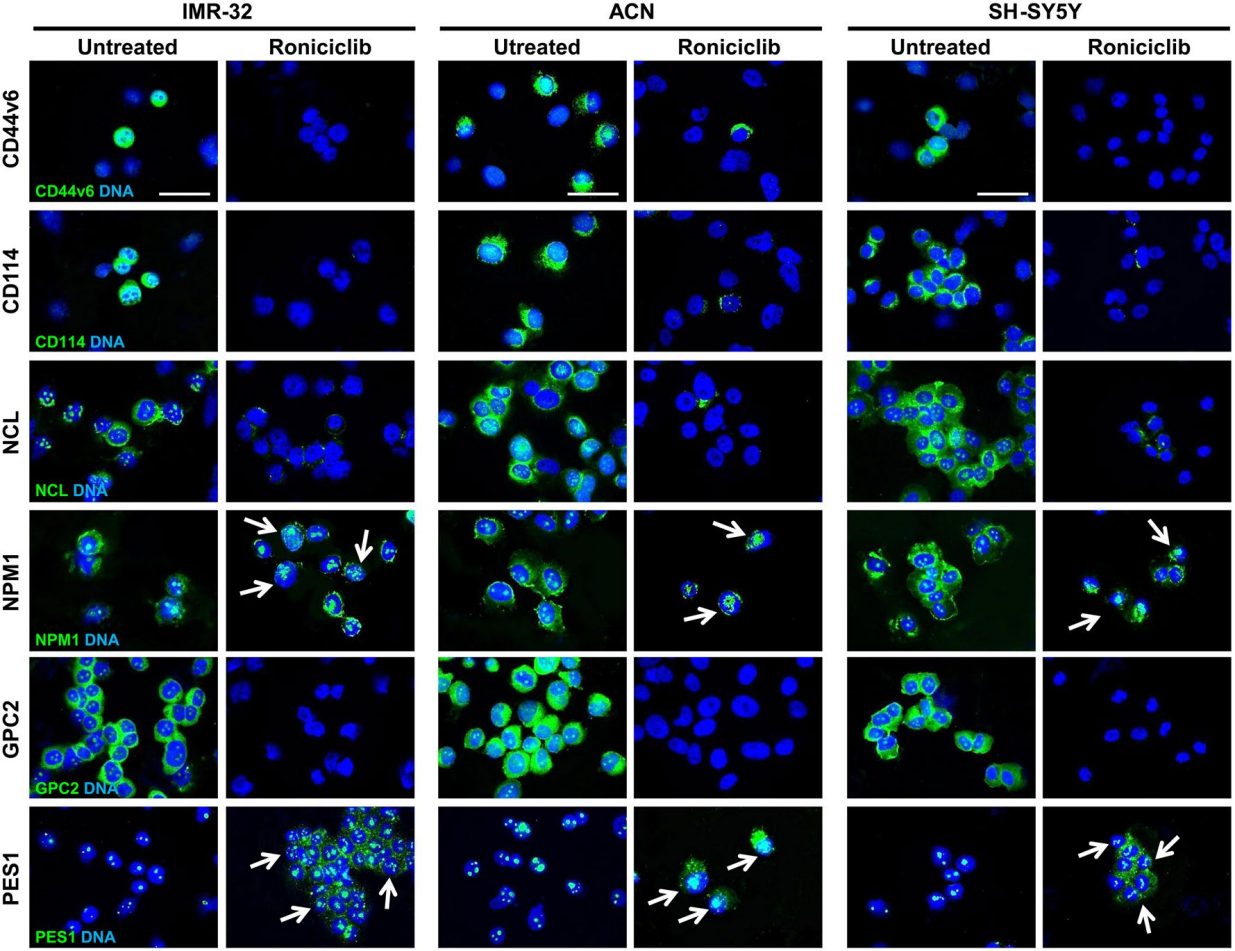
Roniciclib interferes with cancer stem cell markers and induces nucleolar fragmentation in neuroblastoma cells. Immunofluorescence analysis of IMR-32, ACN and SH-SY5Y neuroblastoma cell lines untreated (Roniciclib 0 µM) or treated with Roniciclib 1 µM, 20 µM and 5 µM respectively for 72 h, using anti-CD44v6, anti-CD114, anti-NCL, anti-NPM1, anti-GPC2 and anti-PES1 (all green) antibodies. White arrows indicate nucleolar fragmentation with nucleoplasmic/cytoplasmic redistribution of NPM1 and PES1 proteins. Cells were counterstained with DAPI to visualize nuclei (blue). (Scale bars: 20 µm).

Roniciclib treated NB cells displayed a marked decrease in protein expression for the surface stem cells markers CD44v6, CD114, (Figs. 5 and 6) and for the CD44v6 ligand Osteopontin (OPN) too^55^, more evident in the *MYCN* not amplified cell lines ACN and SH-SY5Y (Fig. 5). As expected, attenuation of stemness in Roniciclib treated NB cells correlated with induction of expression of the neuronal marker microtubule-associated protein-2 (MAP2)^56^, indicative of a differentiated state (Fig. 5); therefore, Roniciclib was capable to determine a clear shift to a differentiated morphology. Moreover, Roniciclib led to the activation of the onco-suppressor protein p53, independent from DNA damage (Fig. 5). Since the iper-activation of the Wnt/β-catenin signal transduction pathway has been implicated in the maintenance or regulation of CSCs self-renewal, in tumorigenesis, and in activation of cancer cell dedifferentiation into CSCs^57,58^, we tested in NB cells the Roniciclib effect on the expression of β-catenin and of the Lipoprotein Receptor-related Protein-6 (LRP6), a transmembrane receptor essential for Wnt-induced signal transduction^59^, observing a strong decrease for both proteins (Fig. 5). All together, these results demonstrate unambiguously that Roniciclib is a negative regulator of stemness.

### Roniciclib induces nucleolar stress in NB cells

**Figure 7:**
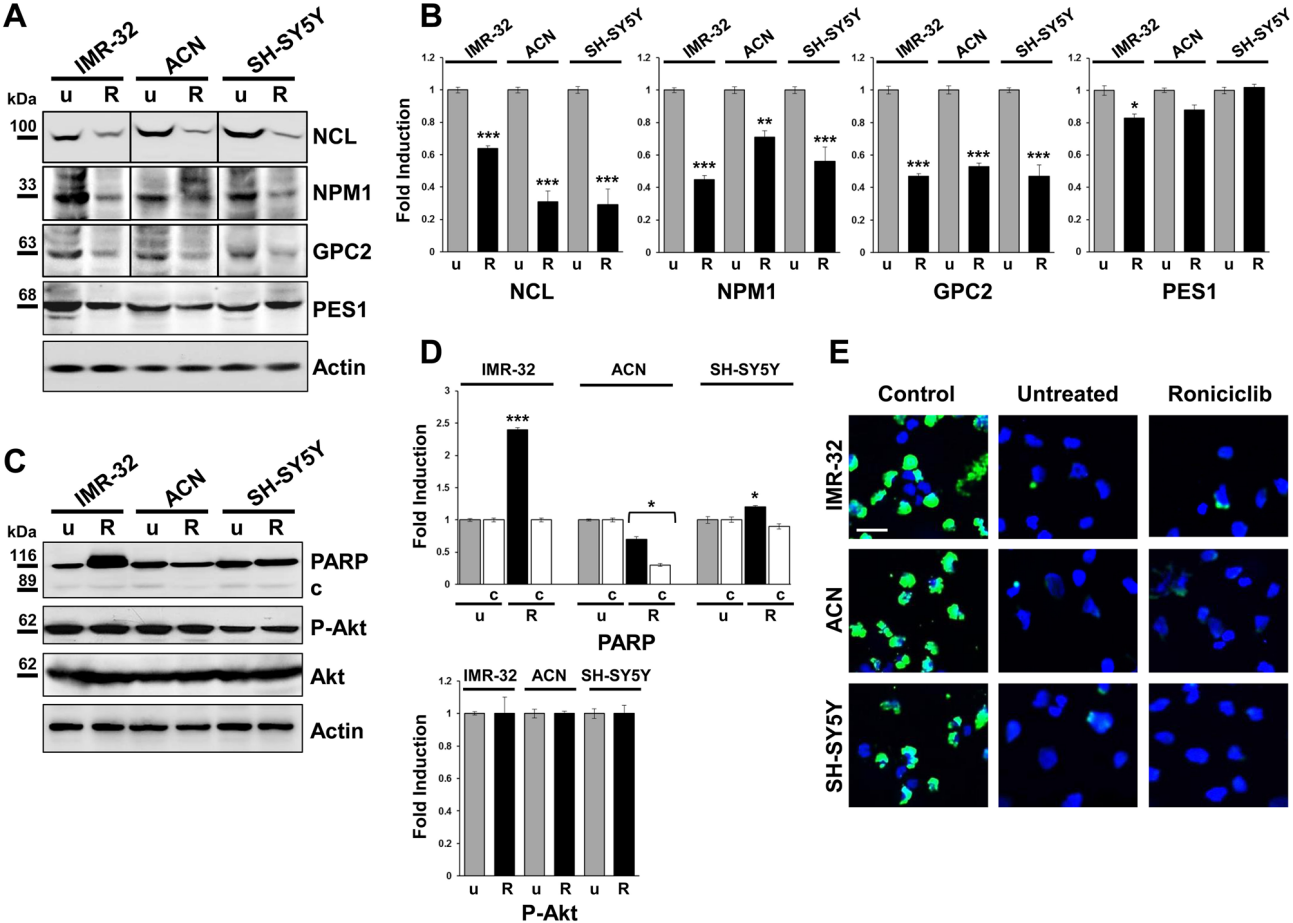
Roniciclib limits nucleolar proteins expression without influencing neuroblastoma cells apoptosis. (**A, C**) Protein lysates from IMR-32, ACN and SH-SY5Y cell lines untreated (u = Roniciclib 0 µM) or treated with Roniciclib (R) 1 µM, 20 µM and 5 µM respectively for 72 h were collected and subjected to Western blot analysis with anti-NCL, anti-NPM1, anti-GPC2, anti-PES1, anti-PARP (c = cleaved PARP), and anti-phospho-Akt (P-Akt) antibodies. Cropped blots are shown here, and black lines indicate where one part of the blot ends and another begins. Supplementary Figure S7, panels 1–2, shows the entire blots images. (**B, D**) Protein levels of the Roniciclib treated cells were quantified by densitometry, normalized to those of the untreated cells (fold induction = 1) and to the content of the loading control protein (Actin or total Akt), then visualized by histograms. Data are representative of three independent experiments ± SD (* *p* < 0.05; ** *p* < 0.01; *** *p* < 0.001). (**E**) Immunofluorescence analysis of IMR-32, ACN and SH-SY5Y cell lines untreated (Roniciclib 0 µM) or treated with Roniciclib for 72 h, using TUNEL staining (green) to reveal DNA strand breaks generated during apoptosis. NB cells pre-treated with DNase I were used as positive controls. Cells were counterstained with DAPI to visualize nuclei (blue). (Scale bar: 20 µm).

Roniciclib treatment caused nucleolar fragmentation (Fig. 6), with the consequent translocation and redistribution of the nucleolar proteins NPM1 and PES1 in most NB cells (ACN 98 ± 4%, IMR-32 88 ± 2%, and SH-SY5Y 84 ± 2% respectively, Supplementary Fig. S3), as well as the almost disappearance of NCL^+^ and GPC2^+^ cells (Fig. 6). NPM1, normally localized in the nucleolus within the nucleus and in the cytoplasm, after treatment with Roniciclib was dramatically re-distributed to the nucleoplasm and in the perinuclear region. We also observed that PES1 translocated from nucleolus to nucleoplasm and cytoplasm (Fig. 6 and Supplementary Fig. S3). The translocation of the nucleolar antigens NPM1 and PES1 is considered the typical hallmark of nucleolar stress^29,60^. Nucleolar stress induced abnormalities in nucleolar structure and function leading to activation of p53 that triggers cell cycle arrest^61,62^. Roniciclib treatment induced nucleoplasmic translocations of nucleolar proteins and induced p53 activation in NB cells (Fig. 5). After Roniciclib treatment, NB cells became less proliferative and more differentiated, the nucleolar stress led to the normalization of the cellular structure and function. As described by Wang et al.^63^, in normal conditions NPM1 is localized in cytoplasm and nucleolus but not in nucleoplasm, and it promotes cell cycle progression particularly when it is located in the cytoplasm^64^. In parallel, by Western blot analysis, we observed in Roniciclib treated NB cells a significant reduction of nucleolar proteins NCL and GPC2 and a less marked depletion for NPM1 and, particularly, for PES1, whose level remained almost unchanged, revealing the Roniciclib effect mainly with their translocation from nucleolus to nucleoplasm and cytoplasm (Fig. 7A, B).

Next, as showed in the Supplementary Figure S4, we confirmed the results observed on cell lines by analyzing the protein lysates extracted from neurospheres cultured under normal conditions or subjected to different Roniciclib concentrations for three days. We found a clear inhibition of expression for the surface stem cell markers CD44v6 and CD114, and for the nucleolar proteins NCL, NPM1 and GPC2, while PES1 showed again a less marked variation. β-catenin and LRP6 were down-regulated too by Roniciclib on NB neurospheres, further supporting the idea of an existing connection among Roniciclib action, stemness depletion and nucleolar stress, which would induce the neurospheres disruption described in Fig. 4.

To test the effects of Roniciclib treatment on cellular apoptosis, we detected by western blotting the expression of the DNA repair enzyme Poly ADP-Ribose Polymerase (PARP) and the amount of protein cleavage^65^. We observed no cleaved PARP in treated NB cells (Fig. 7C, D), but only an augmented level of the uncleaved protein in IMR-32 treated cells, the most sensitive ones, maybe as a way of cellular protection in response to stress^66^. In addition, we showed that Roniciclib did not induce any variation in the RAC-alpha serine-threonine-protein kinase (Akt) phosphorylation, indicating absence of cell proliferation too^67^ (Fig. 7C, D). TUNEL assay staining confirmed that NB cells treated with Roniciclib did not appear to be apoptotic (Fig. 7E). In conclusion, Roniciclib treatment on NB cells, with the dilutions and the exposure time here used, led to morphological changes, inhibited cell growth, but not induced apoptosis.

### Roniciclib reduces tumor growth in a xenograft model of neuroblastoma

**Figure 8:**
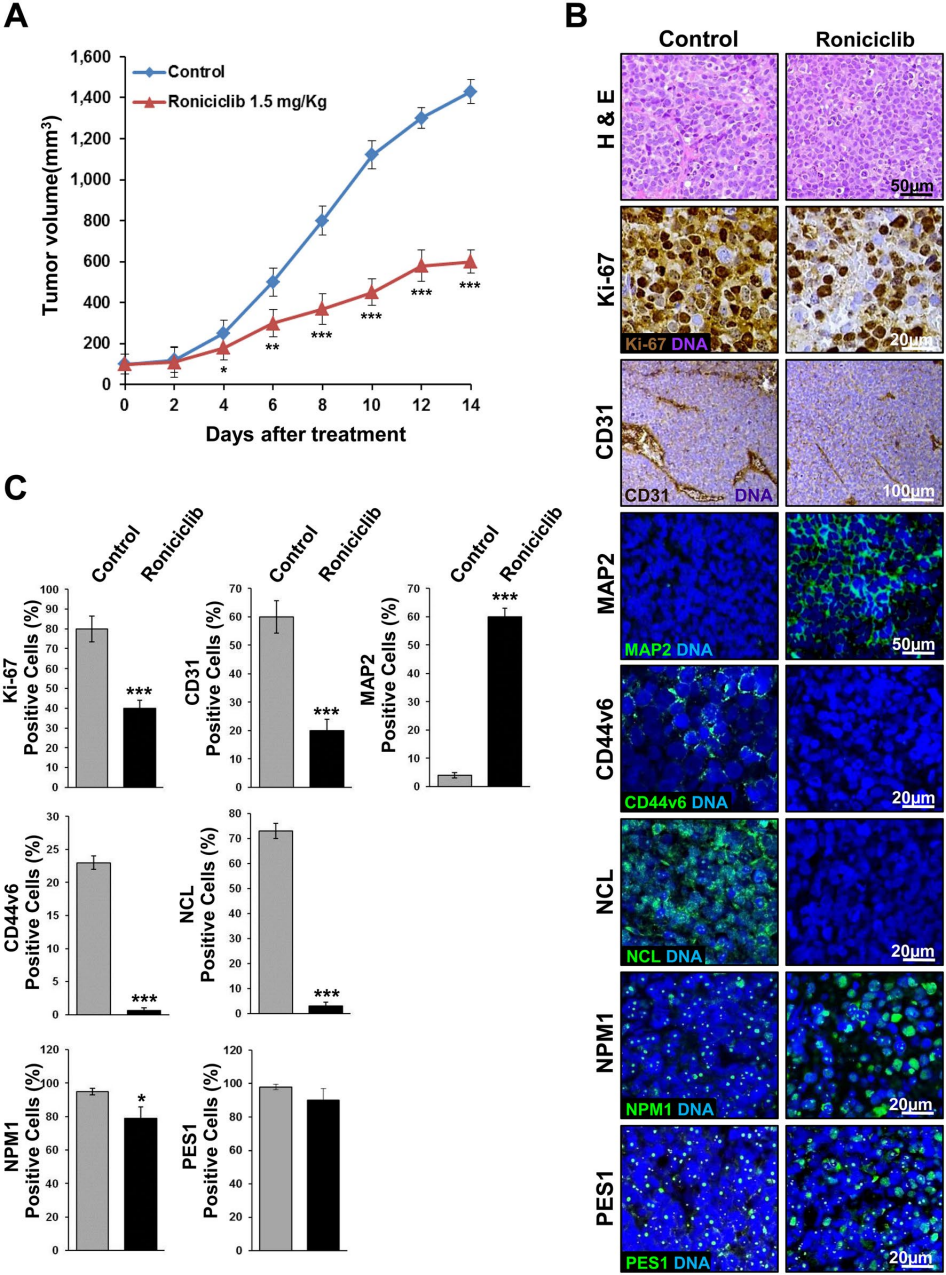
Roniciclib reduces tumor growth in a neuroblastoma xenograft model. 1 × 10^6^ IMR-32 cells were injected in the capsule f the left adrenal gland of nude mice. Mice received oral administration of vehicle control (n = 10) or of Roniciclib (1.5 mg/kg once daily) (n = 10) upon tumor growth. (**A**) Curves indicate tumor volume with vehicle control or with Roniciclib treatment over 14 days. (**B**) In vivo characterization of orthotopic neuroblastoma after 14 days of control vehicle or Roniciclib treatment. For histological analysis, sections were stained with hematoxylin and eosin (H & E). Serial sections were treated by immunohistochemistry with anti-Ki-67 and anti-CD31 (brown) antibodies and by immunofluorescence with anti-MAP2, anti-CD44v6, anti-NCL, anti-NPM1 and PES1 (all green) antibodies. Cells nuclei are counterstained with hematoxylin (violet) or with DAPI (blue). (**C**) The histograms represent the percentage of Ki-67^+^, CD31^+^, MAP2^+^, CD44v6^+^, NCL^+^, NPM1^+^ and PES1^+^ cells. Data are representative of three independent observations ± SD (* *p* < 0.05; *** *p* < 0.001).

In order to investigate the efficacy of Roniciclib as a single chemotherapy agent in a mouse orthotopic neuroblastoma model, IMR-32 cells were implanted in the capsule of the left adrenal gland of nude mice. Tumors were allowed to grow to a palpable volume (~ 100 mm^3^) before the mice were randomized into two groups (10 mice per group) receiving continuous oral administration of Roniciclib (1.5 mg/kg bodyweight once daily)^68^ or vehicle (control) for 14 days. Xenograft tumor volume was significantly reduced by Roniciclib already at day 6 (*p* = 0.03) when compared with vehicle treated mice, and more evidently at day 8 (*p* < 0.0001). At the completion of Roniciclib treatment after 14 days, reduction in tumor volume remained significant (*p* = 0.004) (Fig. 8A). No differences in body weight or side effects were observed in mice during treatment with either Roniciclib or control. To further investigate the effect of Roniciclib, orthotopic tumors were removed from treated or control mice at day 14 for analysis. Ki-67 was used as a proliferation marker, while CD31 and MAP2 were used as markers for vascularization and differentiation, respectively. Treated tumors displayed a significant decrease in Ki-67 and CD31 level, while MAP2 showed stronger expression compared to controls (Fig. 8B, C). We analyzed Roniciclib effect on CD44v6 expression too and we found that positive cells in treated tumors were almost disappeared, indicating an opposite trend in comparison to what we detected previously in post-chemotherapy NB patients samples (Fig. 8B, C and Supplementary Fig. S2A). To assess Roniciclib effect on nucleolar markers in orthotopic tumors, we tested NCL, NPM1 and PES1 proteins. NCL markedly diminished in treated tumors, while NPM1 and PES1, rather than decrease, moved from nucleolus to nucleoplasm or cytoplasm, indicating nucleolar stress activation, as we observed in treated NB cell lines in vitro (Fig. 8B, C). Taken together, our data suggest that Roniciclib treatment in vivo efficiently reduces xenograft NB growth, by influencing different regulatory cell processes, involving, in particular, those related to cell differentiation, to stemness and to nucleolar stress.

## Discussion

Neuroblastoma is an embryonic tumor of the sympathetic nervous system derived from developing precursor cells from neural crest tissue. During development, neural crest cells are the predecessors of the stem cells population that generates neuroblasts, the proposed cells of origin for NB. Despite significant efforts dedicated in advancing new therapies to improve patients’ survival, not all NB cells respond in the expected manner, primarily due to tumor cell heterogeneity^7,8^. To explain this phenomenon, the CSCs hypothesis indicates that tumors contain a subset of tumor-forming and self-renewing stem cells that are less responsive to chemotherapies, and constitute a critical target population for acquisition of drug resistance^69^. Therefore, strategies to prevent CSCs proliferation have been conceived to augment the impact of anticancer therapy. The co-expression of stem cell surface markers like CD44v6 and GPC2, proposed here, and of putative NB stem cell markers, like CD114 or NCL, extends new possibilities to isolate undifferentiated subpopulations from NB. The use of a definitive marker allowing the isolation and characterization of NB stem cell population has been evanescent, most probably a combination of cellular markers will be needed. Notably, in our study nearly 40% of NB cell lines are positive for CD44v6 expression, while less than 2% are CD44v6^+^ in primary NB, and this is because immortalized tumor cell lines are obviously enriched in CSCs. In addition, we found that the percentage of CD44v6^+^ cells increased in NB samples obtained from chemotherapy treated patients who, in large part, suffered a relapse, indicating a possible connection between that antigen and a worse NB patients’ outcome. This observation suggests that CD44v6^+^ cells could be involved in NB drug resistance.

The current therapies generally target highly replicating cancer cells that constitute the bulk tumor mass, but they are not able to eradicate CSCs. For this reason, a focal point in NB research is to better understand cellular heterogeneity within tumors, with a special attention on identifying and targeting CSCs, generally associated with drug resistance. Here, we used Roniciclib (BAY 1000394) a cyclin-dependent kinases (CDKs) inhibitor with potential anti-neoplastic activity. Roniciclib is currently in Phase II of clinical development as a first-line therapy, in combination with chemotherapy, for multiple tumor types refractory to standard therapies^70^. Roniciclib binds to and inhibits the activity of CDK1/Cyclin B, CDK2/Cyclin E, CDK4/Cyclin D1, CDK6/Cyclin D3, and CDK9/Cyclin T1, which are serine/threonine kinases playing key roles in the regulation of tumor cell proliferation and survival. Roniciclib has been shown to induce growth inhibition in NB cells in vitro, and may be considered as a promising novel anticancer agent despite its largely unknown mechanism of action^39^. Here, we demonstrated that Roniciclib acts by inhibiting the expression of LRP6 receptor and β-catenin, two Wnt/β-catenin pathway-correlated molecules, in both NB cell lines and in their related neurosphere derivatives, that could contribute to stemness depletion, inhibition of cell proliferation and impairment of neurospheres formation. Roniciclib successfully reduced cell division and induced cell differentiation as demonstrated by morphology changes (neurite-like growth), and by higher expression of the neuronal differentiation marker MAP2. In response to Roniciclib, the expression of the cell surface stemness antigens CD44v6, CD114, NCL and GPC2 is inhibited in both cell lines and neurospheres, showing a more pronounced reaction to treatment especially for NB CSCs. These results strongly suggest that Roniciclib preferentially suppresses NB stem cells. In addition, it has been demonstrated that inactivation of NCL leads to nucleolar disruption and to cell cycle arrest^71^. It is known that cell division arrest for neuronal cells is the pre-requisite to begin the differentiation process^72^. Therefore, the nucleolar stress induced by Roniciclib contributes to cell cycle arrest and, consequently, to stemness depletion, and all these effects act synergistically to suppress NB tumor growth.

Interestingly, we further demonstrated that Roniciclib activates the nucleolar stress-dependent p53 protein, inducing nucleolar disruption and cell cycle arrest of NB cells. Nucleolar stress is characterized by stressor-induced impairments in nucleolar morphology and function, leading to disturbance in cell homeostasis through activation of p53^37^. It has been proposed that the translocation or redistribution of the nucleolar protein NPM1 is the hallmark of nucleolar stress^28,29^. NPM1 is a key nucleolar protein that can move from nucleolus to nucleoplasm and from nucleus to cytoplasm. Nucleoplasmic translocation of NPM1 is necessary for p53 activation during nucleolar stress. Emerging evidences show that the nucleolus plays a critical role in neuronal development and maintenance, and nucleolar dysfunction has been involved in neurodegenerative diseases^73–75^. Nuclear export of NPM1 to cytoplasm is required for cellular proliferation, while its translocation to the nucleoplasm leads to death through abortive cell cycle induction^76^. Moreover, NPM1 displays anti-apoptotic properties in NB cells^77^ and this could in part explain why, in our experiments with Roniciclib, we clearly notice cell proliferation inhibition without apoptosis induction. The nucleolar stress induced by Roniciclib in NB cells causes the release of NPM1 and PES1 from the nucleolus to the nucleoplasm, suggesting that these two major nucleolar protein components can play a complex role in the regulation of NB cells survival, by modifying their sub-cellular localization. Indeed, it has been described that PES1 together with NCL, are unfavorable prognostic markers of stage 4 NB^25,77^. In addition, we evaluated the association between *NPM1* gene expression with outcome using an RNA sequencing dataset of the public R2 platform. High expression of NPM1 gene was associated with reduced survival of stage 4 NB patients suggesting that it may have an oncogenic role in NB. Over-expression of NPM1, resulting in elevated cytoplasmic localization, has been shown to promote cell transformation^78^. Even though amplification of NPM1 has never been observed in NB, *NPM1* gene is a target of c-MYC, which stimulates NPM1 expression by direct binding to the NPM1 promoter^79^. It has been proposed that over-expression of NPM1 may reinforce the DNA damage response, leading cells to sustain the consequent genomic instability. This would in turn allow tumor cells to select the combined mutations necessary for transformation^80,81^. Finally by employing a preclinical mouse model of NB, obtained by implantation of IMR-32 cells into mice adrenal glands, we demonstrated that after Roniciclib administration, NPM1 and PES1 translocate from nucleolus to the nucleoplasm and cytoplasm in vivo too and, in particular, we confirmed an impressive reduction in tumor growth, indicating promising preconditions for the choice of this drug in NB treatment.

Moreover, the inactivation of Ki-67 and P-Akt along with the absence of a significant amount of cleaved PARP, reveal that through the down-regulation of stemness features and the induction of nucleolar stress, even low doses of Roniciclib can reduce cell proliferation without being toxic and causing cell death and apoptosis.

In conclusion, Roniciclib exerts a peculiar anti-tumor effect on NB cells through induction of cell differentiation and of synergistic nucleolar stress, revealing a novel mechanism underlying Roniciclib-mediated repression of cell growth. The co-expression of surface antigens like the stem cell markers CD44v6 and CD114 together with the nucleolar markers NCL and GPC2, here described, extends new possibilities to isolate undifferentiated sub-populations in NB. In addition, we revealed that elevated levels of NPM1 are correlated with NB patients’ short survival, suggesting this molecule as prognostic marker of NB CSCs as well and a promising target for the treatment of NB.

## Methods

### Cell cultures

Human certified NB cell lines IMR-32, ACN and SH-SY5Y were obtained from ICLC-Interlab Cell Line Collection (San Martino-IST, Genova, Italy). NB cell lines were cultured in Dulbecco’s modified Eagle’s medium (DMEM) High Glucose (EuroClone, Milano, Italy) supplemented with 10% FBS (Gibco, ThermoFisher Scientific, Waltham, MA, USA) and with 1% L-glutamine/penicillin–streptomycin, and maintained at 37 °C under 5% CO_2_ in a humidified atmosphere of 95% air and 5% CO_2_. The genomic identity of each line was regularly established by array-CGH, and cell lines were always tested to certify lack of mycoplasma contamination^50^.

### Neurospheres

Neurospheres formation assay was performed in serum free medium containing half mixture of F12 and DMEM Low Glucose, supplemented with EGF 20 ng/mL, bFGF 40 ng/mL, 2% B27 (Gibco, ThermoFisher Scientific) and 1% l-glutamine/penicillin–streptomycin. Cells were seeded in suspension culture dishes or in standing culture flasks. Starting from the third or fourth day following the cells seeding in serum-free medium, spheres were observed and photographed under the CKX41 phase-contrast microscope Olympus (Tokyo, Japan) equipped with the Altra-20 digital camera, and with the AnalySIS-getIT imaging acquisition software (Olympus). Neurospheres were collected and isolated from single cells for Western blot analysis, by filtering the culture medium with nylon filters (41 µm net) mounted in a Swinnex filter holder (Millipore, Burlington, MA, USA) as described in Palmini et al.^82^. For immunofluorescence analysis, neurospheres were collected, concentrated in little volumes, picked up in drops placed on a slide and fixed.

### Patients and tumor samples

Primary NB human tissue sections analyzed were obtained from 13 patients with metastatic tumor (INSS stage 4) coming from pre-chemotherapy biopsy, and from post-chemotherapy surgical resection (Supplementary Table S1), all diagnosed by the Italian Association of Pediatric Hematology and Oncology (AIEOP). Tumor stage was defined according to the International NB Staging System (INSS)^83^. The post-chemotherapy surgical resections were obtained after induction with rapid COJEC. Rapid COJEC (two courses of carboplatin, etoposide, vincristine; four courses of cisplatin, vincristine; two courses of etoposide, cyclophosphamide) is a time-intensive chemotherapy regimen administered at 10-days intervals^50^. The patients were enrolled in the HR-NBL-1 protocol (SIOPEN)^49^. Informed consent was obtained from all individual participants included in the study. The samples, stored in the BIT-NB Biobank of IRCCS Gaslini (Genova, Italy), are anonymized and the personal data are not disclosed to any researcher. This study was conducted in accordance with the Declaration of Helsinki and approved by the Italian Institutional Ethics Committee (Measure n° 270/17 related to the clinical study protocol IGG-NCA-AP-2016)^50^.

### Roniciclib treatment on cell lines

Roniciclib (BAY 1000394), by AdooQ Bioscience (Irvine, CA, USA), was dissolved in dimethyl sulfoxide (DMSO) at a final concentration of 20 mM and maintained at − 20 °C until used. For drug treatment, NB cell lines were seeded the day before, and Roniciclib was diluted in complete medium to contain < 0.1% DMSO just before use. NB cell lines were then treated with different concentrations of Roniciclib, from 1 to 20 µM for 24, 48 and 72 h at 37 °C to consider the effects on cell growth and survival. Untreated control cells were cultured in the presence of 0.1% DMSO. Cells were every time trypsinized and counted in Trypan blue to evaluate their viability. Roniciclib concentrations that after 72 h gave evident growth effects without toxicity were used for each cell line in next experiments. Cells were observed and photographed under a phase-contrast microscope (Olympus). Neurite-like protrusions observable after Roniciclib treatment are the brief or longer elongations starting from the cell bodies and that appear very thin for IMR-32 and SH-SY5Y cell lines, and thicker for ACN, with star-like shaped cells. Neurite-like elongation in NB cells has been analyzed for the presence of protrusions longer than one cell diameter and the percentages of neurite-extending cells were counted^54^.

### Antibodies

Antibodies anti-human proteins used for Western blot, immunofluorescence and immunohistochemistry were the mouse monoclonal anti-Nodal (ab55676, Abcam, Cambridge, UK), anti-MAP2 (MS-249-R7, ThermoFisher Scientific), anti-PARP1 (sc-8007, Santa Cruz Biotechnology, Dallas, TX, USA), anti-CD44v6 (33-6700, ThermoFisher Scientific), anti-CD31 (M0823, Dako, Agilent Technologies, Santa Clara, CA, USA), anti-Glypican-2 (sc-393824, Santa Cruz Biotechnology), anti-Nucleolin (39-6400, ThermoFisher Scientific), anti-β-catenin (sc-7963, Santa Cruz Biotechnology), anti-LRP6 (sc-25317, Santa Cruz Biotechnology), anti-p53 (sc-126, Santa Cruz Biotechnology), and the rabbit polyclonal anti-N-Cadherin (04-1126, Millipore), anti-Ki-67 (ab833, Abcam), anti-P-Akt and anti-Akt (9271 and 9272, Cell Signaling Technology Inc., Danvers, MA, USA), anti-CD114 (PA5-28988, ThermoFisher Scientific), anti-PES1 (HPA066670, Sigma, St Louis, MO, USA), anti-NPM1 (HPA011384, Sigma), anti-Osteopontin (AB1870, Millipore).

### Immunofluorescence analysis

NB cells were cytospinned and fixed with 4% paraformaldehyde for 20 min, permeabilized in PBS containing 0.3% Triton X-100 for 5 min, and blocked for 30 min at room temperature (RT) with 1% BSA in PBS. Paraffin-embedded tissue sections (4 μm) were deparaffinized, subjected to antigen retrieval in hot 10 mM sodium citrate buffer, permeabilized in PBS containing 0.2% Triton X-100 for 10 min and then blocked with BSA solution for 30 min at RT. Cells or sections were incubated overnight with the primary antibodies at 4 °C or 37 °C. The secondary antibodies conjugated to Alexa 488 (green) or Alexa 568 (red) (ThermoFisher Scientific) were used as recommended by the supplier. Isotype matched non-binding antibodies were utilized in each experiment to avoid non-specific reactivity. Counterstaining of nuclei was performed with DAPI (4′,6-diamidino-2-phenylindole) (Vector Laboratories, Peterborough, United Kingdom). The samples were imaged using the fluorescence microscope Axio Imager M2 equipped with ApoTome System (Carl Zeiss, Oberkochen, Germany)^50^.

### Immunohistochemistry analysis

Paraffin-embedded tissue sections (4 μm) were deparaffinized, subjected to antigen retrieval in hot 10 mM sodium citrate buffer, and then blocked with BSA solution for 1 h at RT. They were incubated with primary antibodies overnight at 4 °C, washed with PBS, incubated with secondary antibodies for 30 min at RT, and developed with DAB reagent (Dako). Sections were counterstained with hematoxylin, dehydrated, and then coverslips were mounted with DPX (Sigma) and slides observed and photographed under the light microscope CX40 Olympus, equipped with the Altra-20 digital camera^51^.

### Western blot analysis

Protein lysates were obtained by lysing cells in Staph-A buffer (1.6 mM NaH2PO4; 8.6 mM Na2HPO4; 1% Triton X-100; 0.1% SDS; 0.1% NaCl; 0.5% NaDoc; 2 mM AEBSF; 20 mg/mL each of aprotinin and leupeptin). Proteins (100 µg) were subjected to SDS-PAGE electrophoresis, transferred to PVDF membrane (Millipore), and then blots were probed with the primary antibodies, followed by the HRP-conjugated secondary antibodies raised in mouse or rabbit (ThermoFisher Scientific). Anti-β-actin antibody (sc-47778, Santa Cruz Biotechnology) was used as loading control. Bands visualization was performed by ECL Select Western Blotting Detection Reagent (Amersham, GE Healthcare, Little Chalfont, UK), then signal intensity was measured by densitometry using Image Lab 6.0 software (ChemiDoc, Bio-Rad, Hercules, CA, USA) and normalized to the loading control^50^.

### Detection of apoptotic cells by TUNEL assay

Apoptotic cells were detected using in situ Cell Death Detection Kit (Roche, Basel, Switzerland) according to the manufacture’s protocol, as described previously^51^. The quantification of apoptosis at single cell level is based on labeling of DNA strand breaks generated during apoptosis (TUNEL technology). DNA breaks are labeled on the free 3′-OH termini with fluorescein-dUTP nucleotides, and visualized by fluorescent microscopy. As positive control, ACN, IMR-32 and SH-SY5Y cells were pre-treated with DNase I (3000 U/ml in 50 mM Tris–HCl, pH 7.5, 1 mg/ml BSA) for 10 min at 15–25 °C to induce DNA strand breaks^51^.

### Mouse model

All procedures involving animals were performed in respect of the National and International current regulations (D.lgs 26/2014 art.23; European Union Directive 2010/63/EU). Experimental protocols were approved by the Italian Institutional Ethics Committee for Animal Care (Measure No. 1033/2016-PR). Mice were athymic nude female (BALB/c-nu/nu) 5–6 weeks old (Envigo, Huntingdon, UK). In all experiments, mice were anesthetized with ketamine (Imalgene 1000, Merial Italia SpA, Milano, Italy), subjected to laparotomy and injected with IMR-32 cell line (1 × 10^6^ cells in 10 µl of saline solution) in the capsule of the left adrenal gland, as previously described^51^. No mice died as a result of this treatment. In the first experiment on five mice, paraffin sections of the orthotopic tumors formed were analyzed by immunofluorescence for the presence of CD44v6^+^/CD114^+^ cells. In the subsequent experiment on 20 mice, once tumor reached an average volume of 100 mm^3^ (about 2 weeks), the animals were randomized to the Roniciclib treatment group (n = 10), or to the vehicle group (control) (n = 10). Roniciclib was orally administered at 1.5 mg/kg bodyweight once daily continuously for 14 days and mice were sacrificed at the end of this period, to analyze tumors. Nude mice are little and almost transparent-skinned animals, so tumor masses were visible, detectable by palpation, and measurable with an acceptable precision. Tumor volume was measured every second day and calculated by the following formula: V = (W^2^ × L)/2) where V is tumor volume, W is tumor width and L is tumor length. The vehicle was 40% Polyethylene glycol 300 (Sigma) and 60% water. The harvested tumors were finally measured with accuracy by a caliper, formalin fixed, and paraffin sections were analyzed by hematoxylin–eosin staining and by immunofluorescence and immunohistochemistry analysis.

### Statistical analysis

Experiments were carried out in triplicate in at least two independent experiments. For all analyses, the significance of differences between experimental samples and controls was ascertained by ANOVA analysis with Bonferroni Multiple comparison Test (* *p* < 0.05; ** *p* < 0.01; *** *p* < 0.001)^50^. The microarray data from the R2 Genomic Analysis and Visualization Platform was analyzed using the R2 web application, which is publicly available at https://r2.amc.nl84. We performed on line Kaplan–Meier analyses and assessment of genes expression comparing different patient subgroups in a large NB cohort (Kokac data set), and we downloaded the resulting survival curves and *p* values obtained with log-rank test.

## Supplementary information

Supplementary Information. (PDF 1340 kb)
