# Supplementary material for: Roniciclib down-regulates stemness and inhibits cell growth by inducing nucleolar stress in neuroblastoma
Source: Sci Rep. 2020 Jul 31;10:12902. doi: 10.1038/s41598-020-69499-6 (PMC7395171; doi:10.1038/s41598-020-69499-6)
Supplement: Supplementary file 1 — Supplementary Information. (PDF 1340 kb) [file 41598_2020_69499_MOESM1_ESM.pdf]

Roniciclib down-regulates stemness and inhibits cell growth by inducing nucleolar stress in neuroblastoma

Marzia Ognibene[1,*] and Annalisa Pezzolo[1,*]

[1] Laboratorio Cellule Staminali Post Natali e Terapie Cellulari, IRCCS Istituto Gaslini, Genova, 16147, Italy

* Corresponding authors: annalisapezzolo@gaslini.org; marziaognibene@gaslini.org

SUPPLEMENTARY INFORMATION

Supplementary Table S1

Clinicopathological characteristics of neuroblastoma patients analyzed for CD44v6+ at pre-chemotherapy biopsy and post-chemotherapy tumor resection.

Patient Number	Stage (INSS)	Therapy Protocol	*MYCN* Status	Age Months	Relapse	Pre-therapy CD44v6+ %	Post-therapy CD44v6+ %
1	4	HR-NBL-1	Amp	7	Y	1.5±0.1	7.5±3
2	4	HR-NBL-1	Not amp	22	Y	1.0±0.2	6.2±1.5
3	4	HR-NBL-1	Amp	9	N	1.9±0.1	19±2
4	4	HR-NBL-1	Amp	43	N	1.8±0.1	9.4±2.1
5	4	HR-NBL-1	Not amp	43	Y	0.8±0.1	6.4±1
6	4	HR-NBL-1	Amp	101	Y	0.7±0.3	6.7±0.9
7	4	HR-NBL-1	Not amp	52	N	1.0±0.2	10.5±0.8
8	4	HR-NBL-1	Amp	65	Y	0.5±0.1	5.2±1.6
9	4	HR-NBL-1	Amp	32	Y	1.0±0.3	9±0.6
10	4	HR-NBL-1	Amp	45	Y	0.6±0.4	6.6±1.2
11	4	HR-NBL-1	Amp	10	N	0.5±0.1	5±3
12	4	HR-NBL-1	Amp	126	Y	0.5±0.2	5.4±1
13	4	HR-NBL-1	Amp	78	Y	0.7±0.2	5.7±2.2

Abbreviations: INSS, International Neuroblastoma Staging System; Amp, amplified; Y, yes; N, no; Data are representative of three independent observations ± SD (p <0.001).

Supplementary Figure S1

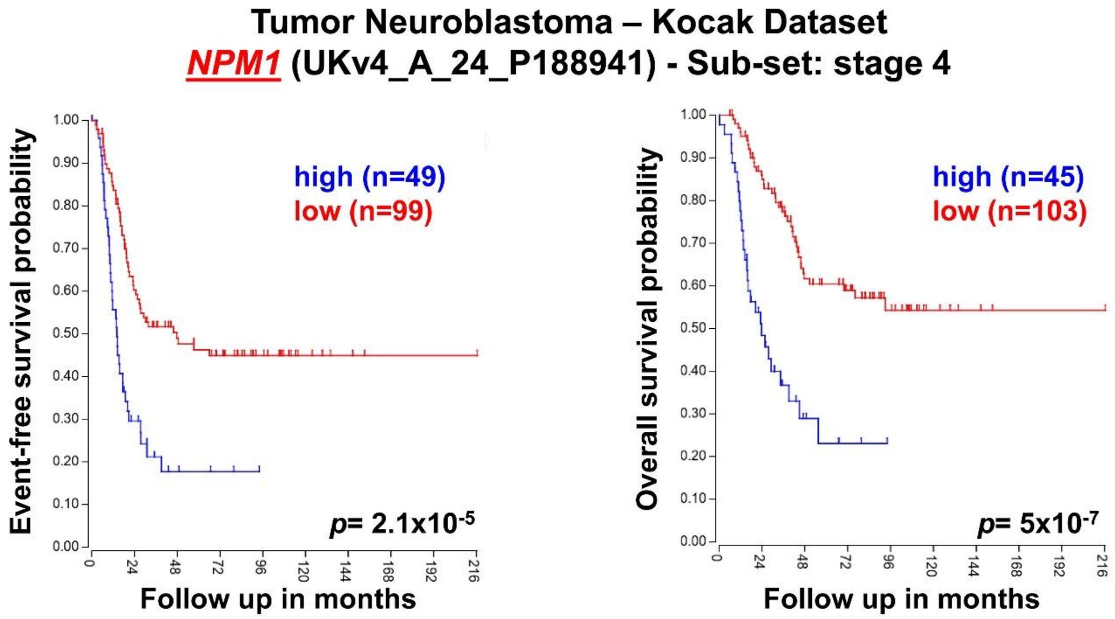

Neuroblastoma stage 4 patient outcomes based on NPM1 gene expression using the neuroblastoma Kocak patients' data-set. Kaplan-Meier analysis and comparison of NPM1 gene expression with patients' outcome. Stage 4 NB patients were divided into high (blue) and low (red) gene expression groups, with patients' numbers in parentheses. The diagrams show the event-free survival curves and the overall survival curves from the Kocak data-set for NPM1 gene expression. *p* values represent log-rank tests.

Supplementary Figure S2

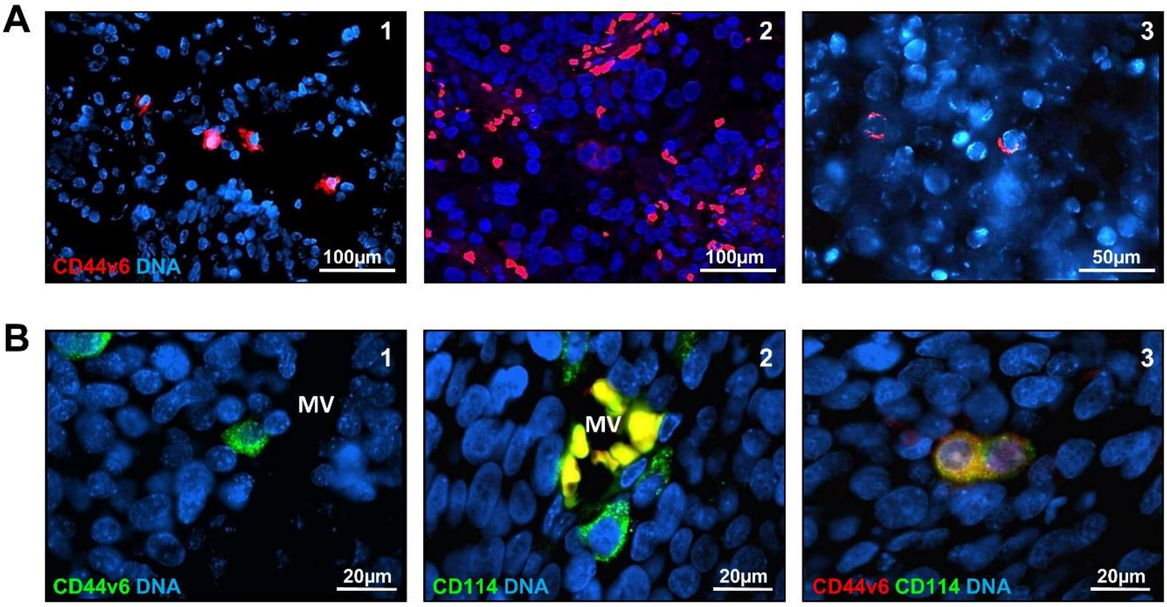

Enhanced expression of CD44v6 in chemotherapy treated and in relapsed neuroblastoma tumors and its preferential localization in perivascular spaces. (**A**) Representative images of immunofluorescence analysis of stage 4 NB sample before (**1**) and after chemotherapy (**2**) and of metastatic bone marrow aspirate from a multi-relapsed patient (**3**), by anti-CD44v6 antibody (red). (**B**) Representative images of immunofluorescence analysis of IMR-32 orthotopic tumors, by anti-CD44v6 (green or red) and anti-CD114 antibodies (green) (**1-2**), with their predominant localization in perivascular spaces, where erythrocytes auto-fluorescence is visible in yellow (MV= micro-vessels). CD44v6 and CD114 antigens co-localize on cell membrane (**3**). Nuclei are counterstained with DAPI (blue).

Supplementary Figure S3

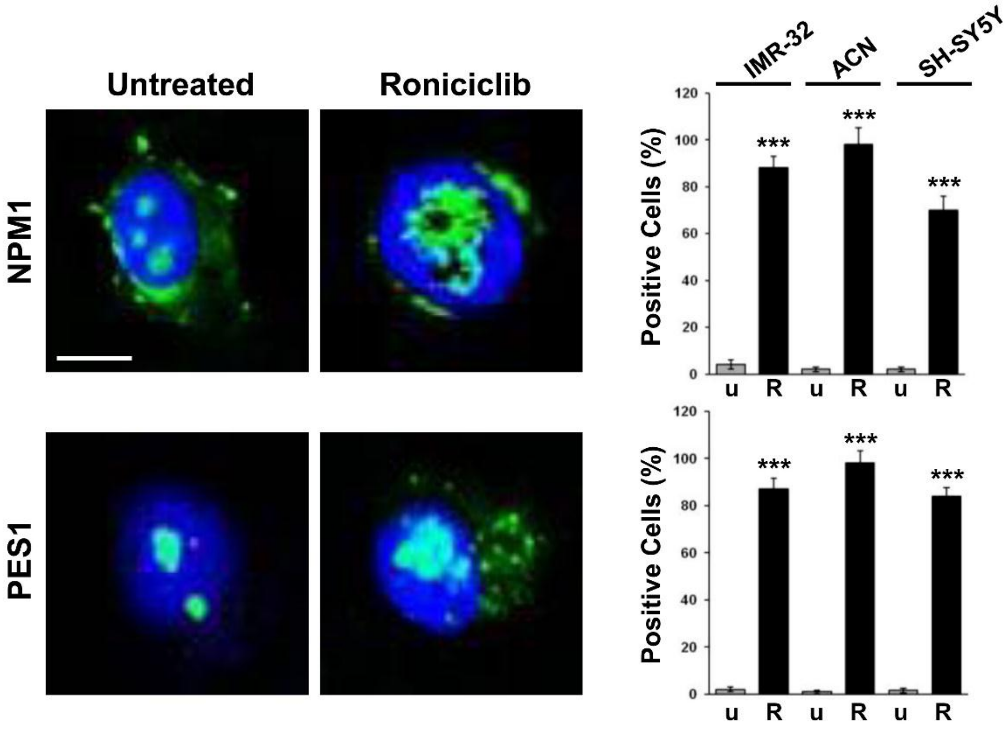

NPM1 and PES1 redistribution after Roniciclib treatment in neuroblastoma cell lines. Enlargement of representative images from Figure 6, as an example of neuroblastoma cells untreated or treated with Roniciclib for 72 h, and analyzed by immunofluorescence. Roniciclib treatment causes nucleolar fragmentation with redistribution of the nucleolar proteins NPM1 to nucleoplasmic and perinuclear localization and of PES1 to nucleoplasm and cytoplasm. Histograms represent the percentage of cells positive for the nucleolar fragmentation for each cell line (u= untreated; R= Roniciclib treated) (Scale bar: 10 µm) (*** p <0.001).

Supplementary Figure S4

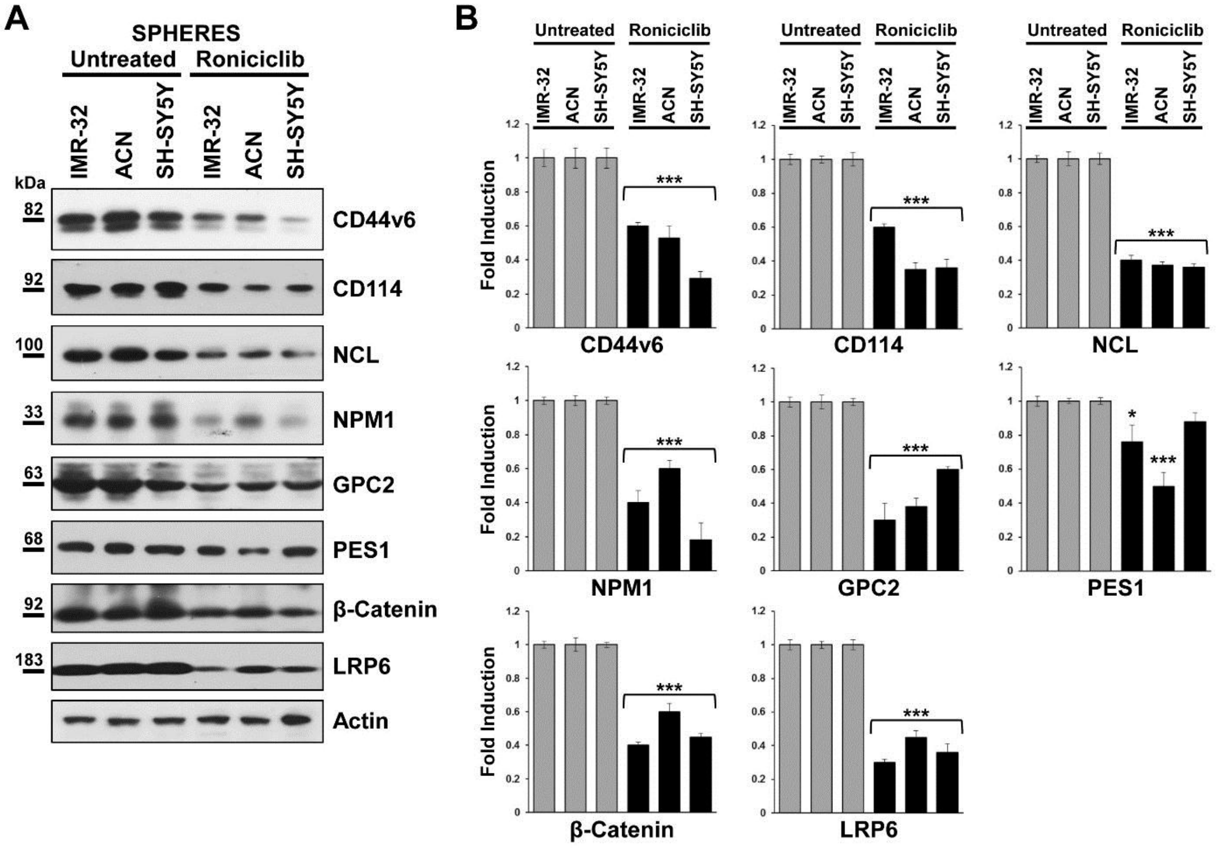

Roniciclib inhibits the expression of cancer stem cells markers, nucleolar proteins and Wnt/β-catenin signal transduction-related molecules in neuroblastoma tumor spheres. (**A**) Protein lysates from neurospheres produced by IMR-32, ACN and SH-SY5Y cells and cultured for three days in serum-free medium without (untreated, Roniciclib 0 µM) or with Roniciclib 1 µM, 20 µM and 5 µM respectively, were subjected to Western blot analysis with anti-CD44v6, anti-CD114, anti-Nucleolin (NCL), anti-Nucleophosmin-1 (NPM1), anti-Glypican-2 (GPC2), anti-pescadillo ribosomal biogenesis factor-1 (PES1), anti-β-catenin and anti-Low density lipoprotein related protein (LRP6) antibodies. Cropped blots are shown here, and black lines indicate where one part of the blot ends and another begins. Supplementary Figure S8 shows the entire blots images (**B**) Protein levels of the Roniciclib treated spheres were quantified by densitometry, normalized to those of the untreated spheres (fold induction = 1) and to the content of the loading control protein (Actin), then visualized by histograms. Data are representative of three independent experiments ± SD (* p <0.05; *** p <0.001).

Supplementary Figure S5 **Uncropped blots for Fig. 1B**

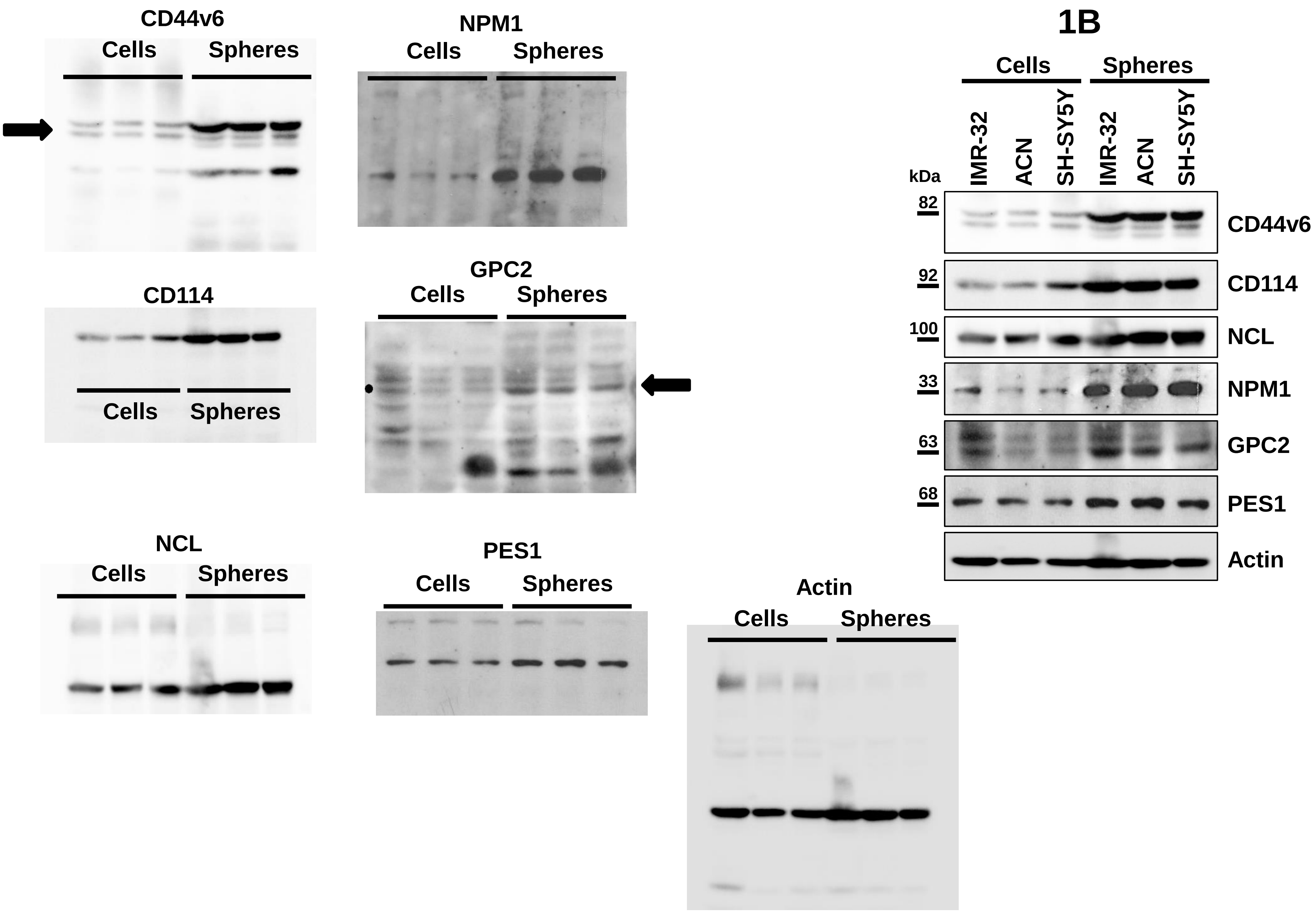

See the statement in the last page of the Supplementary Information file

Supplementary Figure S6 - panel 1 Uncropped blots for Fig. 5A

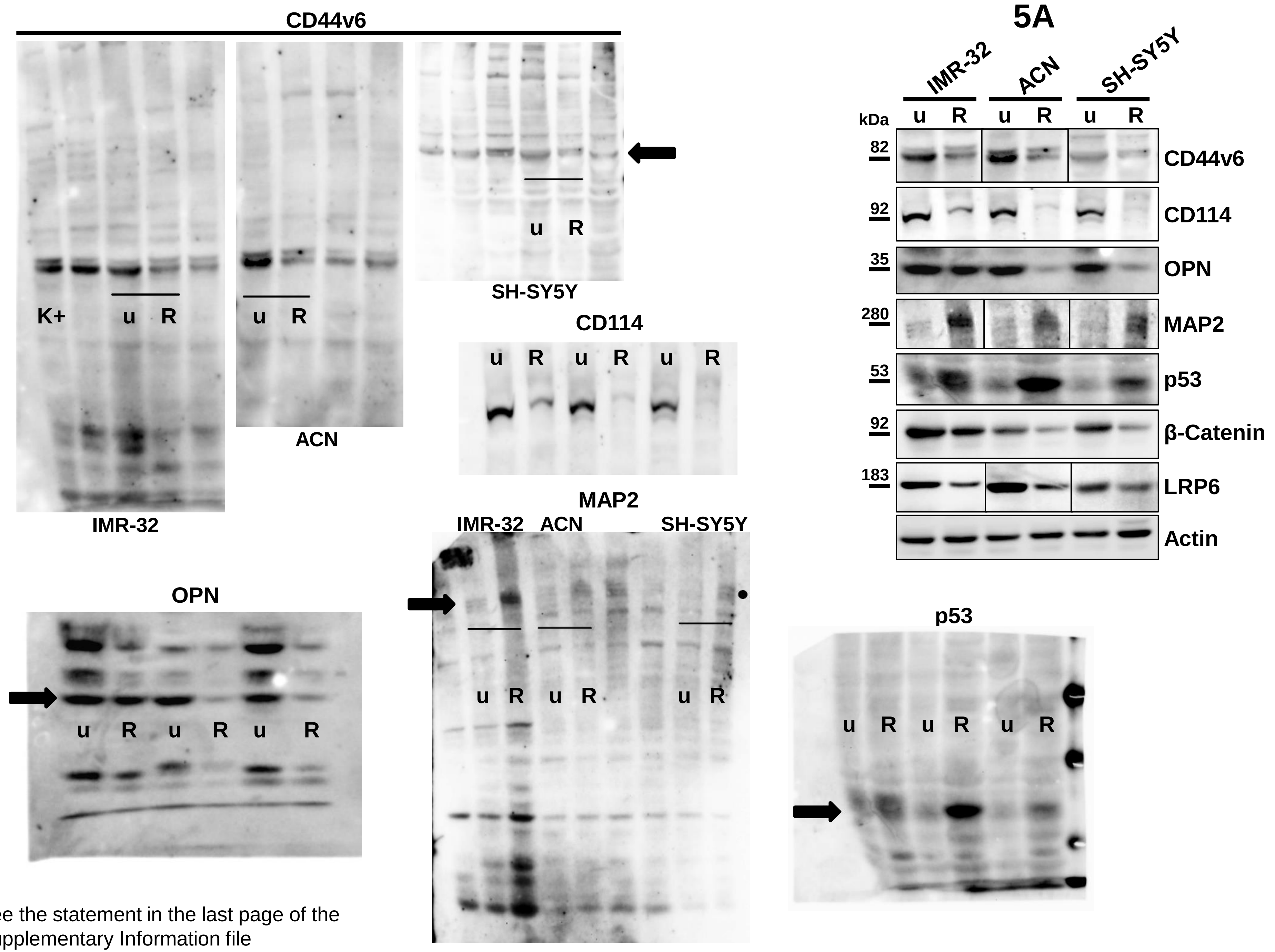

See the statement in the last page of the Supplementary Information file

Supplementary Figure S6 - panel 2

Uncropped blots for Fig. 5A

β-Catenin

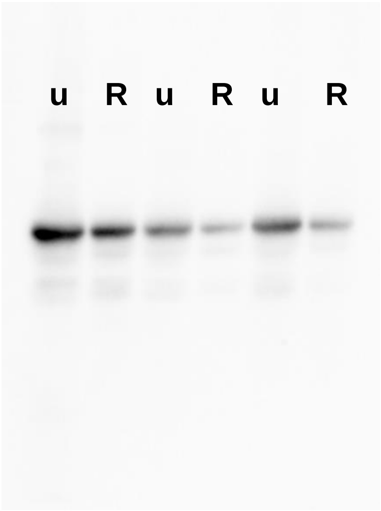

LRP6

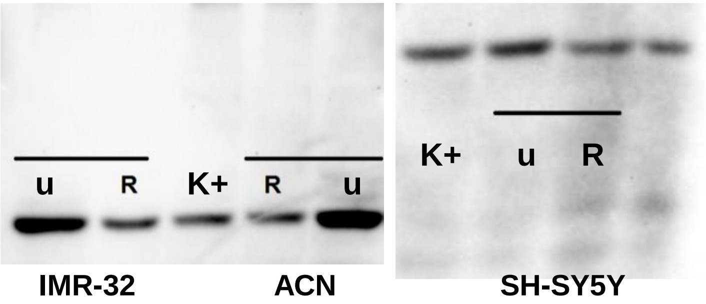

Actin

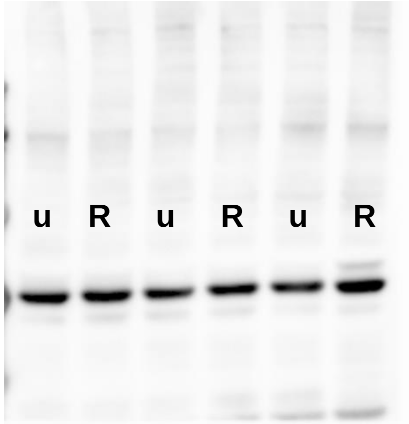

5A

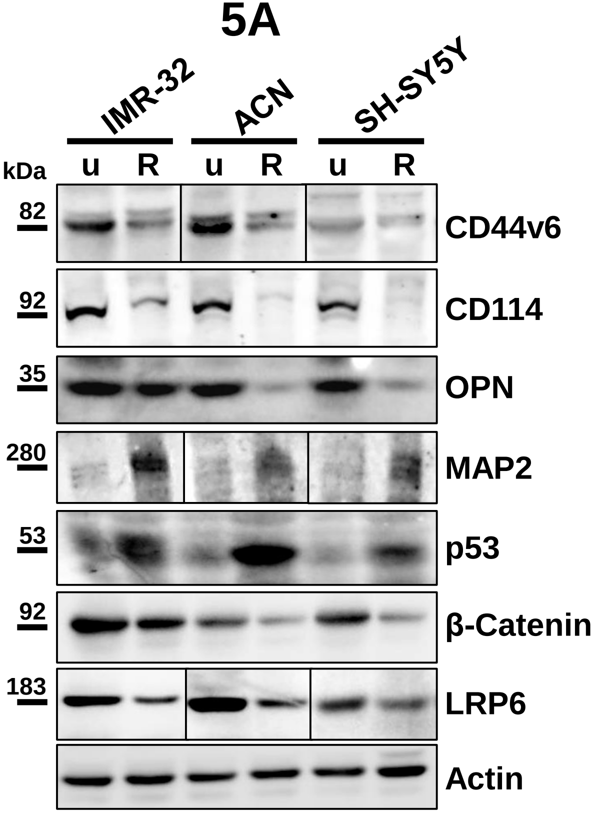

See the statement in the last page of the Supplementary Information file

Supplementary Figure S7 - panel 1

Uncropped blots for Fig. 7A

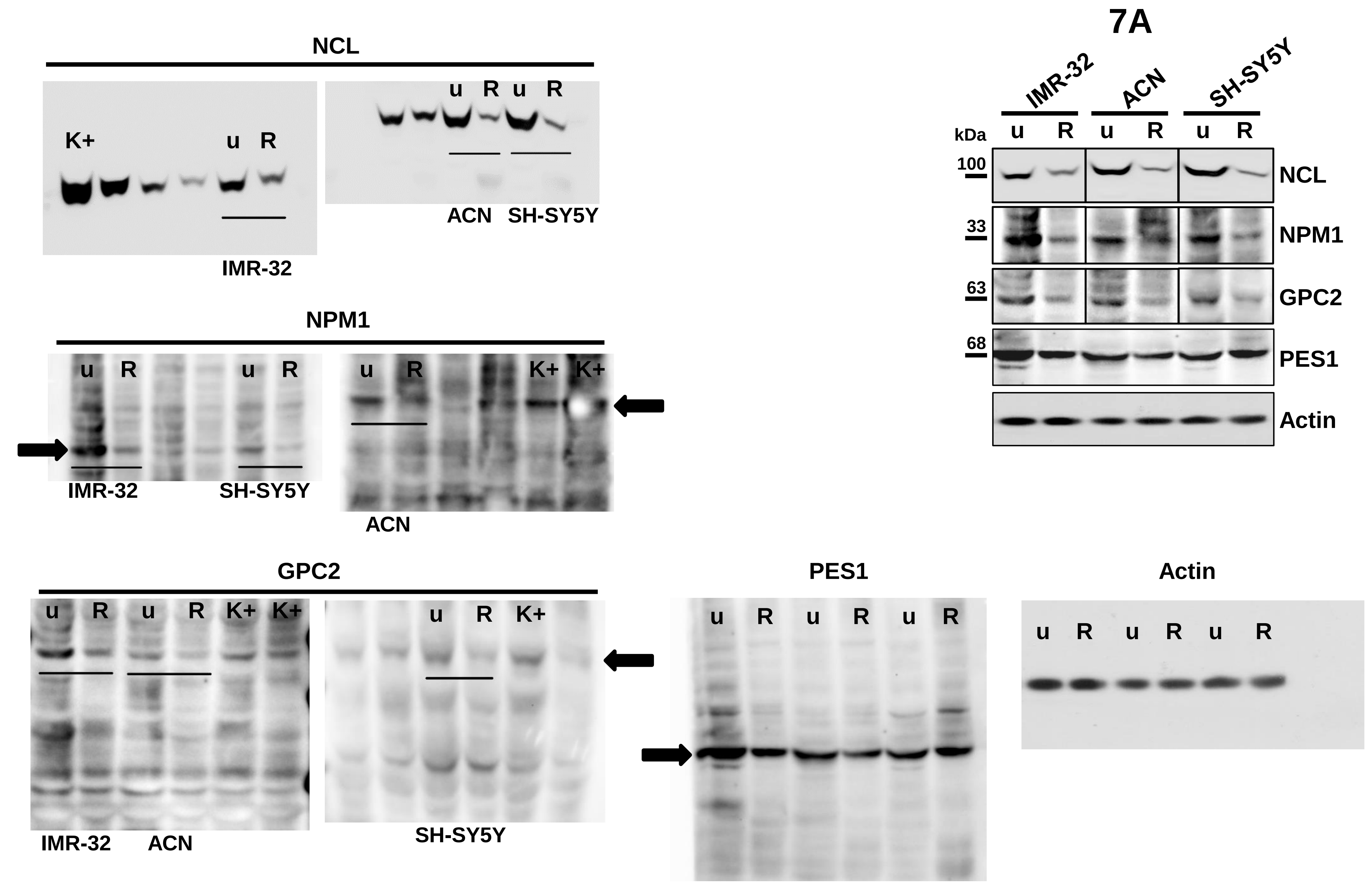

See the statement in the last page of the Supplementary Information file

Supplementary Figure S7 - panel 2 Uncropped blots for Fig. 7C

PARP

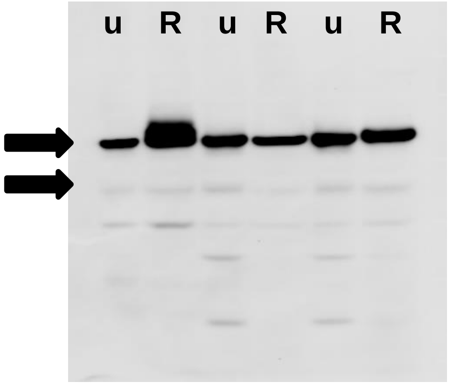

P-Akt

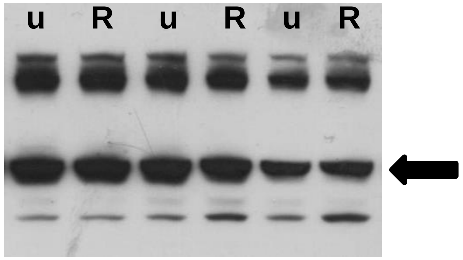

Akt

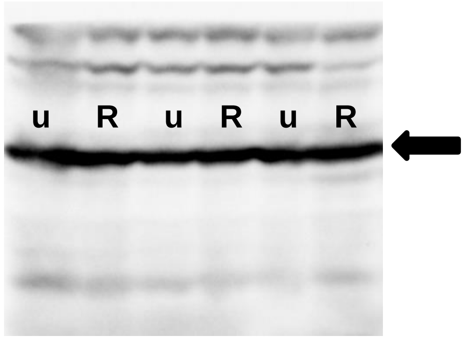

Actin

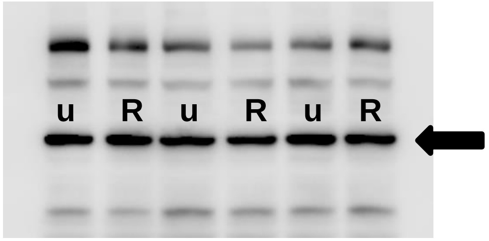

7C

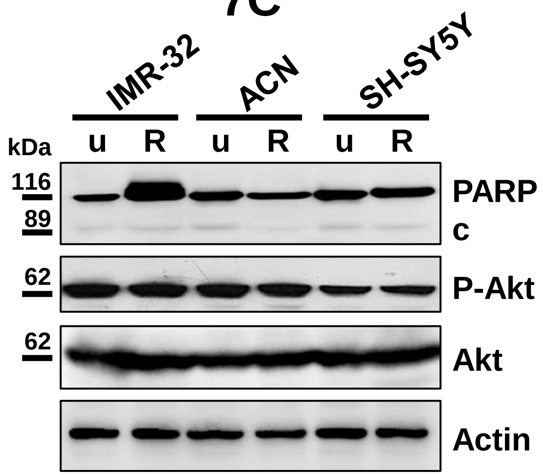

See the statement in the last page of the Supplementary Information file

Supplementary Figure S8

Uncropped blots for Supplementary Fig. S4A

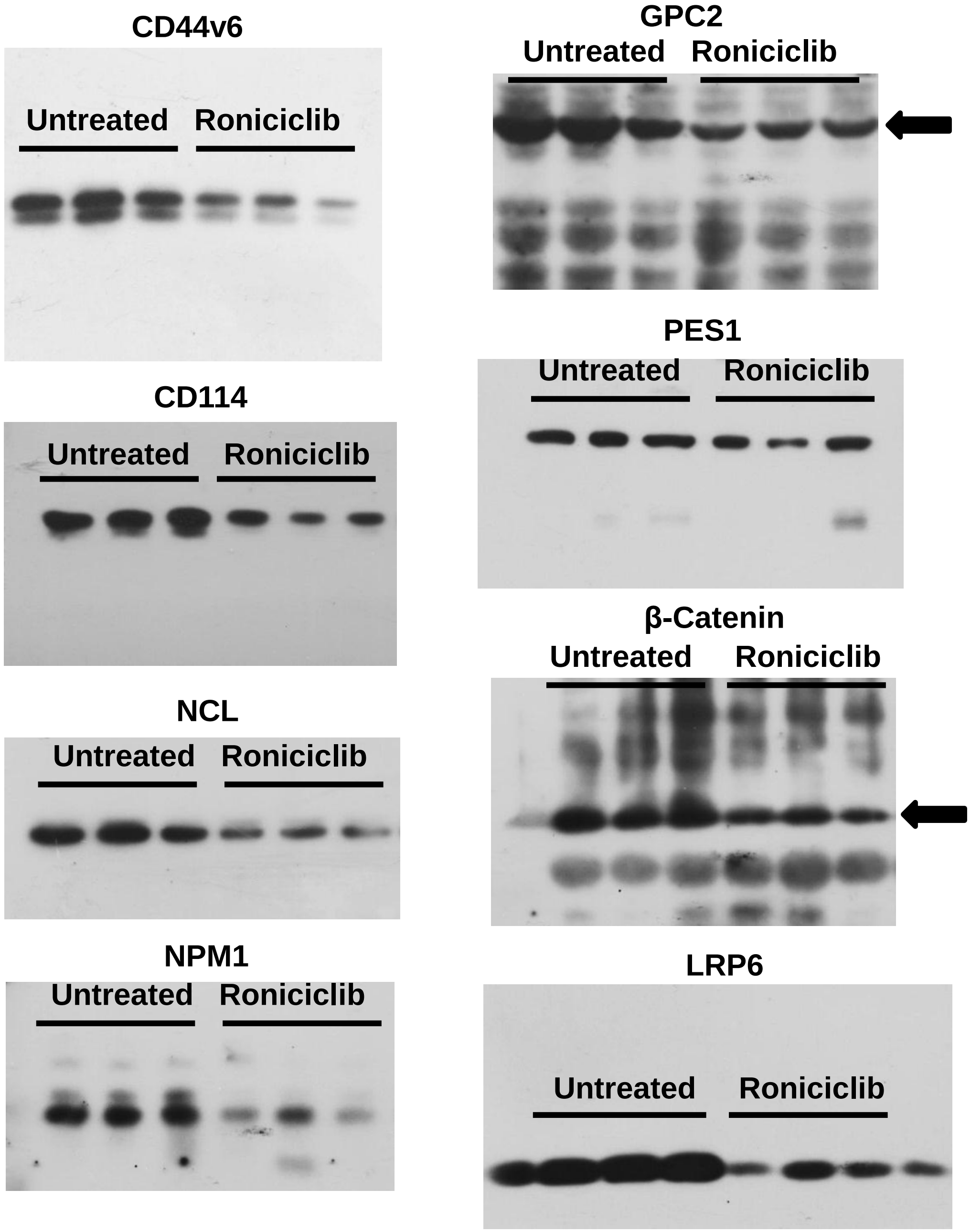

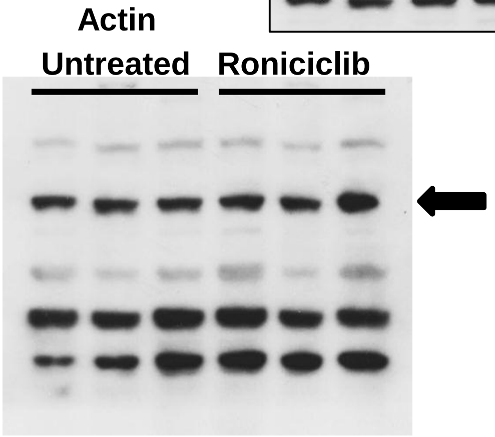

Supplem. S4A

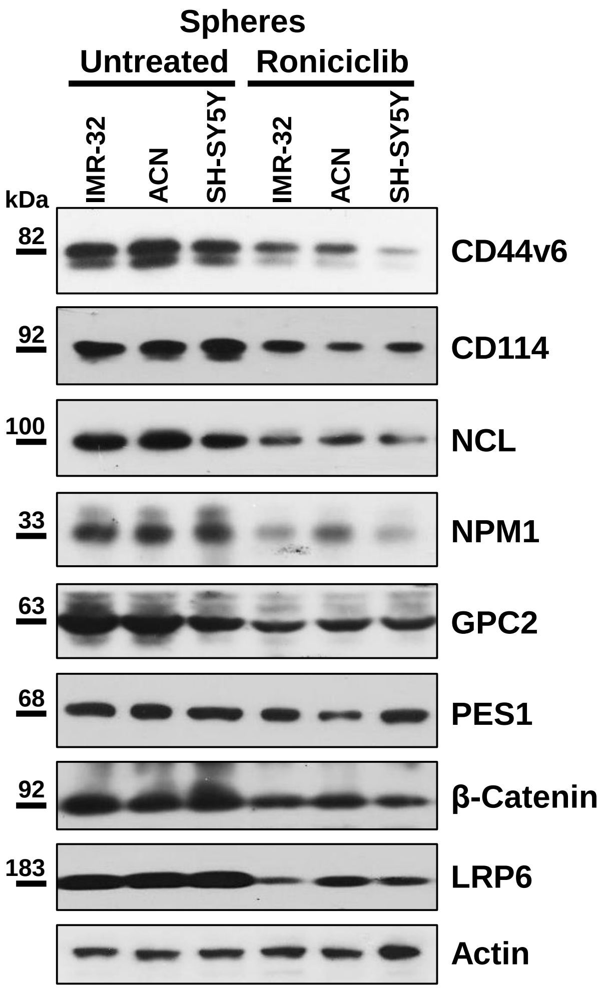

See the statement in the last page of the Supplementary Information file

Roniciclib down-regulates stemness and inhibits cell growth by inducing nucleolar stress in neuroblastoma

Marzia Ognibene and Annalisa Pezzolo

Statement about Western blots images presented in the manuscript

The western blots figures in the manuscript present only the specific bands for every protein, in order to have clear and more manageable images.

Figures panels containing western blots derive from the assembly of different images obtained from different blots or different times of exposure of the same blot, for the three cell lines used: only one exposure time image for every blot has been here collected. Cropped blots are indicated by black lines delimiting where one blot ends and another begins. Beside every group of original images in Supplementary Figures S5, S6, S7 and S8, we have put the corresponding figure of the manuscript, to easily check each blot composing the figure itself.

We use a pre-stained protein marker that leaves very faint bands, and it is undetectable in chemiluminescence, so we have to retrace it every time on the membrane with a pencil. After evaluating the right bands merging the colorimetric image containing the protein ladder with the chemiluminescence image, we usually save the best images to be used for the manuscript without maintaining the retraced markers on them.

Positive controls (indicated as K+ in the Supplementary Figures) were protein lysates from available cell lines suggested by our antibodies data-sheets or by the web site www.proteinatlas.org. They lack in blots probed with antibodies used previously, for which we already knew the specific pattern in the NB cell lines used, so, for homogeneity, we did not show any positive control in the manuscript figures, while they are visible in the Supplementary figures, and they were: for CD44v6, older and tested lysate from IMR-32 cell line; for GPC2, older lysates from IMR-32 and SH-SY5Y cell lines; for NCL and LRP6, lysate from HeLa cell line; for NPM1, lysates from HeLa and MCF-7 cell lines.

When possible, we stripped the membranes and we probed them again with other antibodies, or, alternatively, we cut one membrane at different levels of the protein marker, in order to obtain different fragments to probe with different antibodies at the same time. Consequently, for each Western blot panel there is only one image for the anti-actin antibody, or we have chosen one anti-actin re-probing representative for all the blots in the panel.

We performed three independent experiments for each western-blot figure, where every blot in the figure came from the same experiment, therefore from the same protein lysate for each cell line: the figures prepared for the manuscript show the best experiment among the three.

Since images were all obtained with a Bio-Rad ChemiDoc instrument, sometimes the area of interest was directly cropped and saved, if nothing else specific or peculiar was detectable on the blot.
